# Application of Hyperspectral Imaging and Generative Adversarial Network for Powdery Mildew Severity Detection on Melon Leaves

**DOI:** 10.3390/plants15172639

**Published:** 2026-08-28

**Authors:** Zhiqi Hong, Chu Zhang, Li Fang, Hongxia Ye, Hui Fang, Yong He

**Affiliations:** 1 College of Biosystems Engineering and Food Science, Zhejiang University, Hangzhou 310058, China; hzq@zju.edu.cn (Z.H.);; 2 The Rural Development Academy & Agricultural Experiment Station, Zhejiang University, Hangzhou 310058, China; 3 School of Information Engineering, Huzhou Normal University, Huzhou 313000, China; 4 Institute of Plant Protection and Microbiology, Zhejiang Academy of Agricultural Sciences, Hangzhou 310021, China; 5 Institute of Vegetable Science, Zhejiang University, Hangzhou 310058, China

**Keywords:** melon, powdery mildew, deep convolutional generative adversarial network, conditional deep convolutional generative adversarial network, hyperspectral imaging, convolutional neural network

## Abstract

Powdery mildew in melon reduces fruit quality and yield, and may cause plant death. Accurate severity diagnosis supports disease control, resistance evaluation, and precision management. This study applied hyperspectral imaging to assess disease severity in two cultivars (Zhetian 105 and Zhetian 501) under five stress levels. To explore the feasibility of generative models for spectra generation for disease severity classification, three generative models (Conditional Generative Adversarial Network (CGAN), Deep Convolutional Generative Adversarial Network (DCGAN), and Conditional Deep Convolutional Generative Adversarial Network (CDCGAN)) were used to generate different numbers of samples for each stress level. To evaluate the generated data quality, classification models (logistic regression (LR), support vector classification (SVC), eXtreme Gradient Boosting (XGBoost), and Convolutional neural network (CNN)) models were built under three conditions: using real training samples to predict real test samples; using generated samples to predict the real test samples; and using real training samples with generated samples to predict real test samples. The results showed that DCGAN and CDCGAN can generate the samples close to the real training samples. Classification models using generated samples, as well as the combination of real training samples and generated samples, showed increase in the classification performance on the real test samples. The different numbers of generated samples did not show specific patterns on the prediction performance. Some models using generated samples and real training samples with generated samples could improve the prediction accuracy up to 10% compared with the models using real training samples. Class-wise analysis indicated that not all prediction performance of different classes increased. The overall results indicated that generative adversarial networks have the potential to generate reflectance spectra of different melon cultivars under varying disease severities. More real samples and generation strategies are needed to better improve the generated data quality.

## 1. Introduction

Melon (*Cucumis melo* L.) is a globally popular fruit, highly valued for its distinct flavor and texture. As a thermophilic crop, it is extensively cultivated in temperate and tropical regions. However, melon production is frequently constrained by pests and pathogens, which compromise both fruit quality and yield. Driven by shifting dietary preferences, the greenhouse cultivation of off-season produce has gained significant popularity. However, suboptimal management in these controlled environments often exacerbates disease pressure. Powdery mildew, a predominant disease in melons, primarily infects leaves, petioles, and stems, with initial symptoms manifesting on the foliage. This pathogen severely impairs plant development, leading to substantial economic losses through reduced yield and quality and, in severe cases, plant mortality [1].

Accurate detection of powdery mildew on melon leaves is a prerequisite for precision disease management. Traditionally, detection has relied on visual diagnosis by experienced farmers or agricultural technicians based on field symptoms. However, these conventional methods are highly subjective and typically only allow for a rough estimation of disease severity and distribution. In contrast, advanced laboratory techniques, such as PCR assays and microscopic observation [2,3,4], can accurately identify the pathogen. Nevertheless, the high costs, stringent equipment requirements, and specialized operational expertise associated with these methods make them impractical for large-scale screening. Consequently, the application of modern sensing technologies for rapid, non-destructive, and high-throughput detection of plant diseases has emerged as a prominent research hotspot.

Visible and near-infrared (Vis-NIR) spectroscopy is a mainstream high-throughput plant phenotyping technique [5] and has been widely investigated for plant disease detection [6]. It detects disease type and severity by sensing disease-induced changes in leaf biochemical constituents. Hyperspectral imaging simultaneously acquires spectral and spatial information [7,8]. It records a spectrum for each pixel, thus capturing spectral data across the entire detection area. Compared with point-measurement Vis-NIR spectroscopy, hyperspectral imaging provides richer and more comprehensive information. Both Vis-NIR spectroscopy and hyperspectral imaging have been extensively applied to plant disease detection, including the detection of infection, classification of disease types, and assessment of severity [9,10,11]. Hyperspectral plant disease detection is not only a spectral classification task, but can also be regarded as an important component of intelligent agricultural sensing and data-driven decision systems. In practical disease management, model outputs are expected to support disease control, cultivar resistance evaluation, and precision management. However, when training samples are limited, imbalanced, or insufficiently representative, purely data-driven models may suffer from insufficient robustness and generalization. Therefore, expert experience, disease grading criteria, and domain constraints can provide biologically meaningful labels and prior constraints, thereby improving model reliability.

In plant disease detection research based on spectral data, supervised modeling is the most commonly used analytical approach. Conventional machine learning methods, including PLS-DA, SVC, Random Forest, ANN, and XGBoost, have been widely applied in diagnosing whether different crops are infected, identifying disease stress types, and assessing disease stress severity [9]. In plant disease diagnosis, achieving higher diagnostic accuracy is one of the key focuses of current research, and applying innovative algorithms is a potential means to improve accuracy. Deep learning is one of the most widely used methods in data analysis today. It possesses a powerful self-learning ability for features and can mine deep features from data. As a reliable modeling method, deep learning has become a mainstream data analysis approach in plant disease detection [12]. Although the strong feature learning capability of deep learning enables research under few-shot [13] or even zero-shot [14] scenarios, existing studies still rely on datasets composed of a large number of samples. In the current research, deep learning algorithms have been extensively studied in spectral-based plant disease detection and have achieved promising results [15,16].

In spectral-based diagnosis of plant disease stress, many studies are conducted through artificial inoculation in greenhouses or growth chambers, where researchers often encounter the problem of unbalanced and small sample sizes across different categories. Class imbalance can cause the model to be biased towards the majority classes during modeling; if the disparity in sample size is too large, it may make the minority classes unpredictable or increase the probability of misclassification [17,18]. How to balance the classes while increasing the number of samples to improve modeling performance and robustness is a challenging problem. In such limited-sample modeling scenarios, reliable label definition is particularly important. Disease severity labels determined based on expert experience and disease grading criteria can reduce label uncertainty and provide a more reliable basis for the subsequent evaluation and selection of generated samples. With the development of deep learning, generative artificial intelligence has become a hot topic in the field of artificial intelligence. By constructing appropriate models, it is possible to generate relevant content such as text, images, videos, and audio [19]. In the domain of spectral data analysis, spectral data generation is one of the main research topics in generative artificial intelligence [20].

Generative Adversarial Network (GAN) and its variants are currently one of the main methods for generating spectral data based on generative artificial intelligence [20]. For GAN-based data generation methods, the most important goal is to generate data that meet practical requirements. Recent generative learning methods have been widely used for data augmentation and reconstruction tasks. In addition to conventional GANs, several GAN variants have been proposed to improve generation controllability, training stability, and sample quality. For example, DCGAN introduces deep convolutional structures into the generator and discriminator, improving feature learning and generation stability [21]. CGAN incorporates class labels as conditional information to guide the generation process and produce samples with specific category attributes [22]. CDCGAN further combines the convolutional architecture of DCGAN with the conditional constraints of CGAN, making it suitable for class-controlled generation tasks [21,22,23]. ACGAN introduces an auxiliary classifier to enhance class-conditional generation [24], while WGAN and WGAN-GP improve adversarial training stability by modifying the distribution distance metric and regularization strategy [25]. Meanwhile, diffusion models have become another important family of generative models. Denoising diffusion probabilistic models and score-based diffusion models formulated through stochastic differential equations learn data distributions through progressive noising and reverse denoising processes [26,27]. Recently, diffusion models have been applied to plant disease image augmentation [28], agricultural near-infrared spectral data augmentation [29], hyperspectral image restoration [30], and broader reconstruction tasks.

For spectral data, based on its principles, specific wavelength bands are related to certain corresponding chemical components. Therefore, during the generation process, the quality of the generated spectra is crucial, and it is necessary to generate spectra that are similar in shape yet distinct to meet various research needs. In GAN-based spectral generation, improving the quality of the generated spectra is a main research focus at present [23,31,32,33]. In the field of spectral-based plant disease diagnosis, GAN-based methods have also achieved numerous research outcomes [32,34].

In this study, hyperspectral imaging was used to detect powdery mildew severity on melon leaves. Disease severity was assessed on two susceptible melon varieties. The leaf samples across different severity levels were limited and class-imbalanced. To explore the feasibility of GAN-based methods to generate spectra for disease severity classification, spectral curves for leaves of the two varieties at different disease severities were generated using three typical generative models: CGAN, DCGAN, and CDCGAN. CGAN and DCGAN represents two different types of commonly used GAN, and CDCGAN is the combination of CGAN and DCGAN. Different numbers of spectra (100, 200, and 300) were generated. The real samples were randomly split into the training and test sets, and the spectra generation were conducted based on the real training set. Then, logistic regression (LR), SVC, XGBoost, and CNN models using real samples, generated samples, and real samples with generated samples as a training set were built, and the real test set was used to evaluate the model performances and quality of the generated samples.

## 2. Results

### 2.1. Spectral Characteristics

In this study, the essential goal was to evaluate the performance of different GAN-based methods for spectra generation; the real samples of Zhetian 105 and Zhetian 501 were randomly split into the training and test sets at a ratio of 1:1 to ensure that there were more samples for testing. The training set samples were used for data generation and corresponding analysis, and the test set samples were used to evaluate the performance of the classification models and generated spectra. Table 1 shows the number of samples in the training and test sets of Zhetian 105 and Zhetian 501. Healthy samples and those with severity levels one, two, three, and four were assigned class labels 0, 1, 2, 3, and 4, respectively.

**Figure 1:**
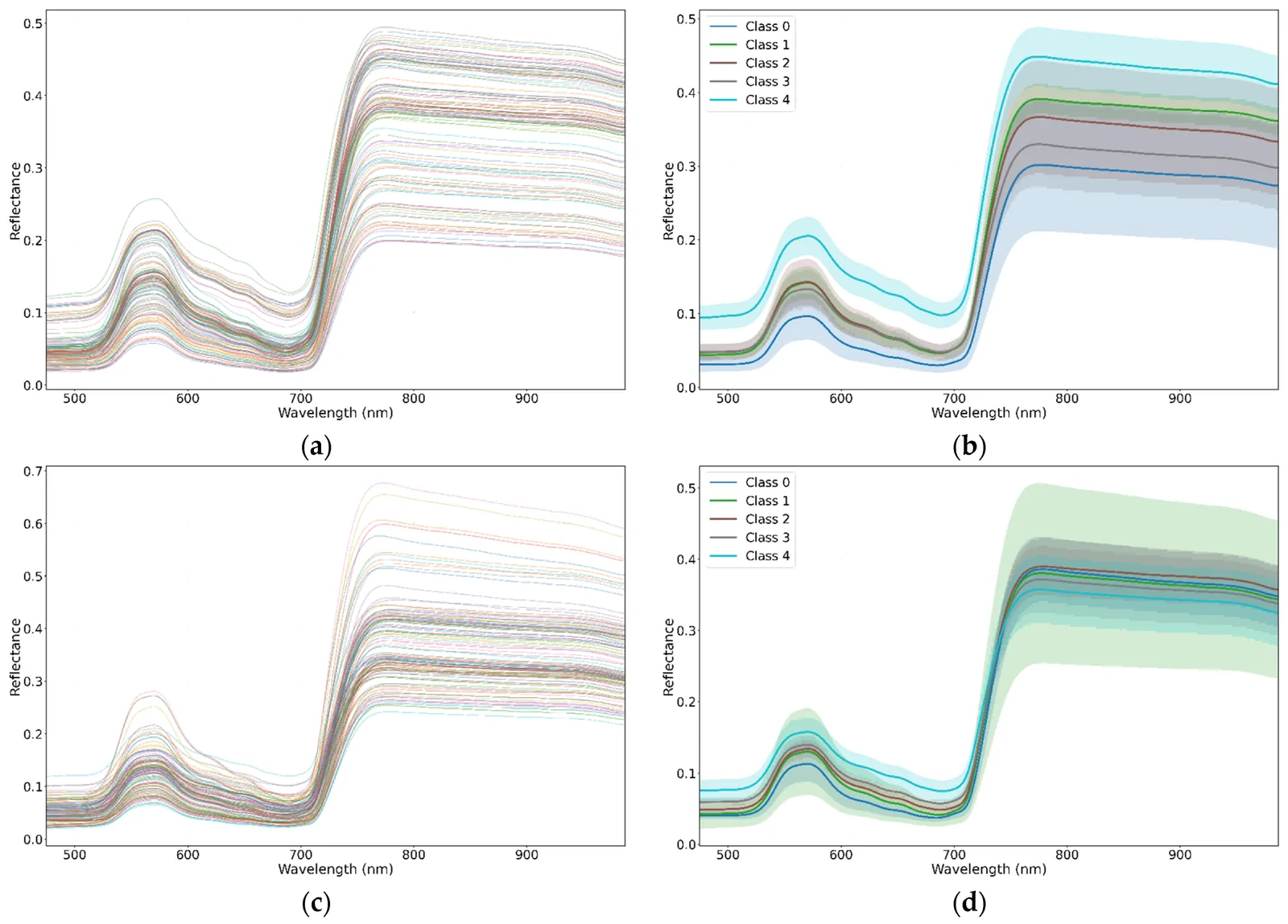
(**a**) Spectral curves of all samples of Zhetian 105 under varying disease stress levels; (**b**) average spectral curves and standard deviations of samples of Zhetian 105 under different disease stress conditions; (**c**) spectral curves of all samples of Zhetian 501 under varying disease stress levels; (**d**) average spectral curves and standard deviations of samples of Zhetian 501 under different disease stress conditions.

Figure 1a,b show the reflectance spectra of leaves in the training set (healthy and with varying disease severity) of the Zhetian 105 variety, as well as the average reflectance spectra and standard deviations for each disease severity level. Figure 1c,d present the corresponding data for the Zhetian 501 variety. The average spectra indicate that, although the spectral trends across different disease severity levels are similar, certain differences in reflectance spectra are still observable. For melon leaves of different varieties, the spectra under varying disease severity also exhibit some differences, without a fully consistent trend. The standard deviation plots reveal overlaps among the spectra of leaves with different disease severity levels.

### 2.2. Classification Results Based on Real Samples

An attempt was firstly conducted to evaluate the performance of the models established using real training set samples to predict the real test samples. The essential goal was to evaluate the performance of different GAN-based methods for spectra generation, and the samples were randomly split into the training and test sets at a ratio of 1:1, as presented in Table 1. The classification results of are shown in Table 2.

As shown in Table 2, the optimal performance for disease severity classification of Zhetian 105 was obtained by the CNN model. Poor performance was obtained by the LR and XGBoost models. For disease severity classification of Zhetian 501, the CNN model obtained good performance, with a classification accuracy over 80% for training and test sets. Poor performance was obtained by the LR and XGBoost models. Although the results were not quite good, these results indicated the potential of disease severity classification of the two melon varieties. It should be noted that overfitting existed in Table 2; this could be attributed that the essential goal of this research was to explore the feasibility of GAN-based methods to generate samples for disease severity classification, and the improvements on the training and test performance were used to evaluate the generated data quality. Thus, the real samples were randomly split into the training and test sets, without considering the sample distribution within one class and the overfitting issue.

### 2.3. Data Augmentation Study

In this study, DCGAN, CGAN, and CDCGAN were employed to generate reflectance spectra of melon leaves at different disease severities. The spectral differences among leaves with varying disease severity arise primarily from changes in leaf physicochemical properties. Accordingly, the generation of one-dimensional spectral data must preserve consistency in both spectral shape and reflectance magnitude, while also reproducing the characteristic spectral distributions associated with each disease severity level. Since the number of real training samples is small, the number of generated samples of each class was set as 100, 200, and 300 to explore the influence of the number of generated samples.

#### 2.3.1. Analysis of Generated Spectral Profiles

**Figure 2:**
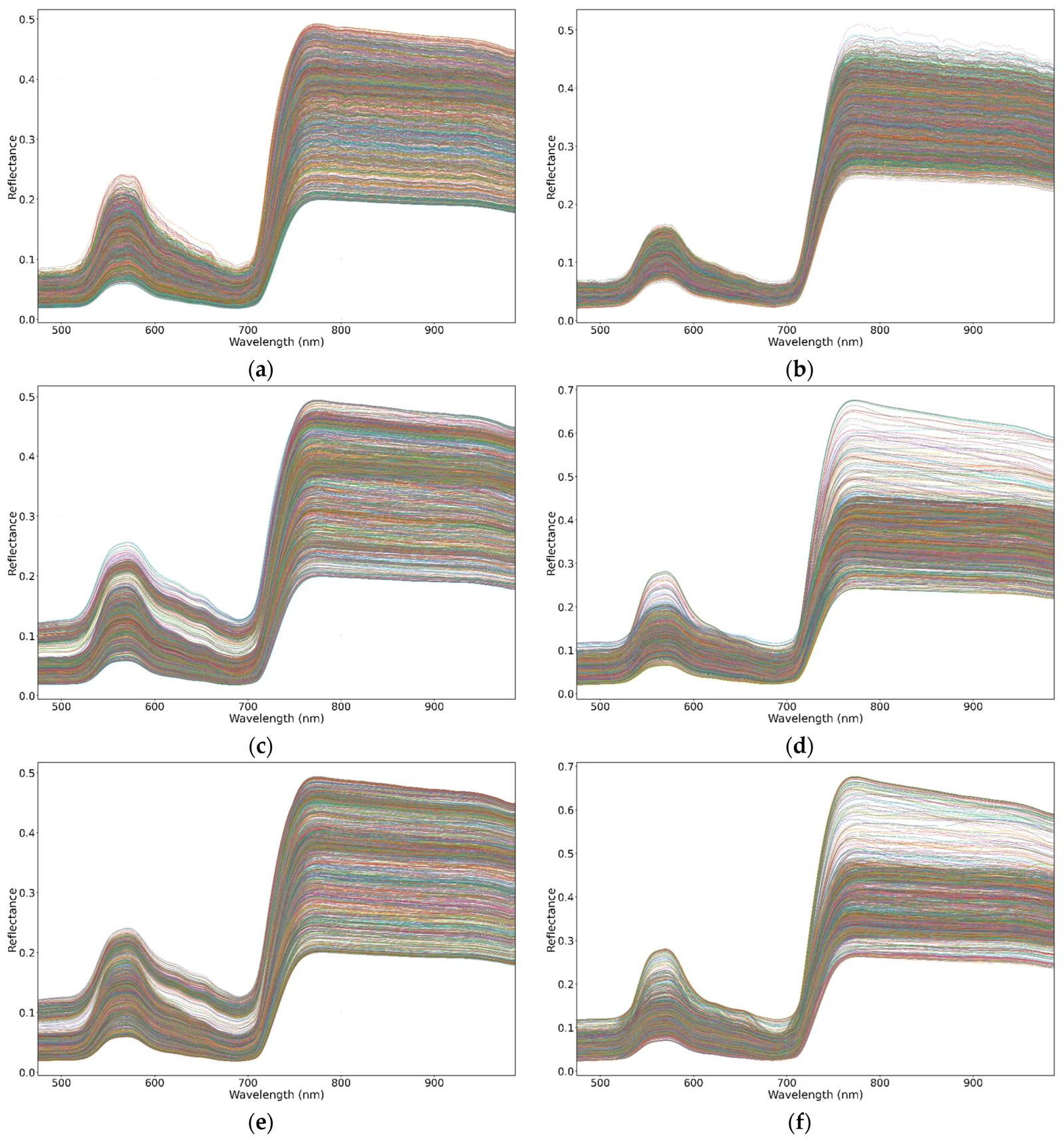
Spectral curves of different disease severity levels generated by different generative adversarial networks (300 generated spectra of each class): (**a**) spectra generated by CGAN for the Zhetian 105 variety; (**b**) spectra generated by CGAN for the Zhetian 501 variety; (**c**) spectra generated by DCGAN for the Zhetian 105 variety; (**d**) spectra generated by DCGAN for the Zhetian 501 variety; (**e**) spectra generated by CDCGAN for the Zhetian 105 variety; (**f**) spectra generated by CDCGAN for the Zhetian 501 variety.

When CGAN was employed, class labels were introduced, enabling the simultaneous generation of spectra for five classes (disease severity levels). However, the spectral curves generated by CGAN in this study exhibited noticeable spike noise, which needs to be further investigated. Figure 2a,b shows the 300 generated spectra of each class. As shown in Figure 2a,b, although the waveforms are highly similar and the generated spectra reflect class information, the noise may compromise the final generation quality.

When generating spectra with DCGAN, the model lacks class information, so spectra for each disease severity level had to be produced separately. The spectra of different disease severities generated by the various generative adversarial networks for the two varieties, Zhetian 105 and Zhetian 501, are presented in Figure 2c,d. DCGAN generated spectra for each class independently but did not incorporate class labels, making it impossible to assign class identities to the generated spectra. Nevertheless, it produced spectra that closely resemble the real data and are free of spike noise, as illustrated in Figure 2c,d. In contrast, although CGAN incorporated class labels, the generated spectra contained pronounced spike noise and required further smoothing. Therefore, this study combined the strengths of DCGAN and CGAN by adopting CDCGAN for spectral data generation. The spectra produced by CDCGAN, as shown in Figure 2e,f, are visually highly similar to the spectra of real samples. Since the spectra generated by CGAN showed noises, these spectra were not used for further analysis. The spectra generated by DCGAN and CDCGAN were used for further analysis.

In addition to spectral profiles, the spectral angle mapper (SAM), Wasserstein distance (WD) and root mean square error (RMSE) were also used to evaluate the different numbers of generated spectra by DCGAN and CDCGAN. SAM was calculated using the pairwise spectra from real training samples and generated samples from the same class. WD computed the overall distribution of real training samples and generated samples of each class. RMSE was calculated using the mean of real training samples and the mean of generated spectra of each class. Tables S1 and S2 show the average values of SAM, WD, and RMSE of the generated sample with real training samples for Zhetian 105 and Zhetian 501, respectively. For Zhetian 105, the SAM, WD, and RMSE values of the spectra generated by CDCGAN are generally comparable to those produced by DCGAN. Overall, across different number of generated samples, the mean SAM of the CDCGAN-generated spectra is slightly lower than that of the DCGAN-generated ones, while the mean WD and RMSE are slightly higher. For different spectral categories, the three metrics are close with certain variations, yet no distinct pattern is observed. For Zhetian 501, similar conclusions can be drawn: the SAM values are comparable, and the CDCGAN-based spectra exhibit a marginally lower average SAM but marginally higher average WD and RMSE compared with the DCGAN-based spectra. The metric values for different categories also show proximity with minor discrepancies, without any discernible regularity. As presented in Tables S1 and S2, for both varieties, the overall generation quality of the synthetic spectra is good according to the low SAM, WD, and RMSE values.

#### 2.3.2. t-SNE Analysis of Generated Data

In addition to spectral profiles, SAM, WD, and RMSE, t-SNE was used to visualize the distribution of real and generated samples of the five classes. t-SNE is a nonlinear dimensionality reduction method designed to map high-dimensional data into a low-dimensional space while preserving local neighborhood structures. The algorithm optimizes the positions of low-dimensional embeddings by minimizing the Kullback−Leibler divergence between the pairwise similarity distributions of the high-dimensional space, represented by a probability distribution, and those of the low-dimensional space, computed using a Student’s t-distribution. The resulting low-dimensional representation is typically visualized as a two-dimensional scatter plot, enabling researchers to intuitively analyze the clustering patterns of the data. Prior to t-SNE visualization, all features were standardized using StandardScaler to eliminate the influence of varying feature scales and numerical ranges. Subsequently, the t-SNE output dimensionality was set to 2, with the perplexity parameter set to 30 and the maximum number of iterations set to 1000. The low-dimensional embedding was initialized via principal component analysis. The learning rate was configured as “auto”, allowing the algorithm to automatically determine its value based on the sample size. The random seed was fixed at 42 to ensure reproducibility across runs.

**Figure 3:**
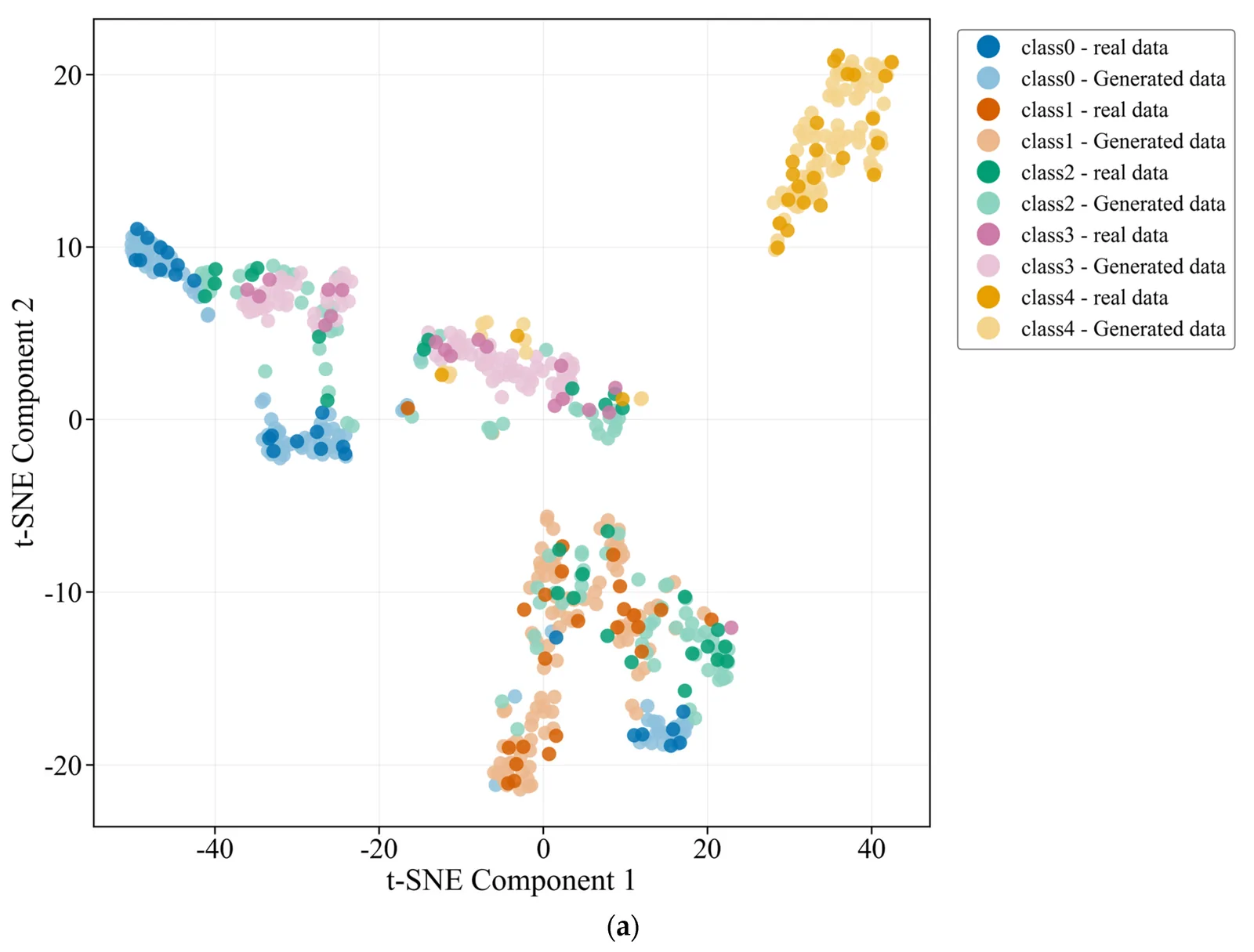

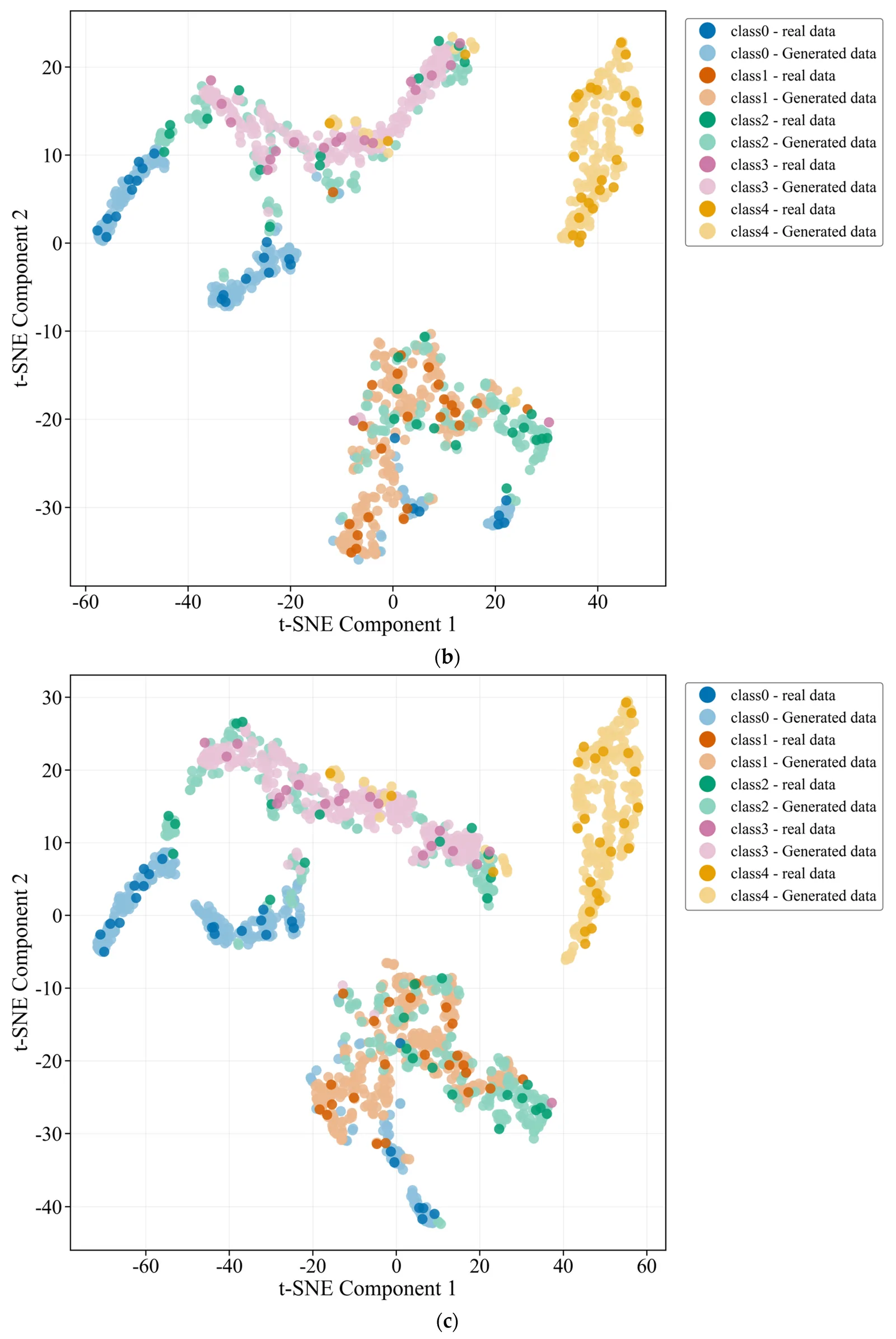
t−SNE visualizations comparing the generated data with the real data for the melon variety Zhetian 105 under DCGAN: (**a**) 100 generated spectra; (**b**) 200 generated spectra; (**c**) 300 generated spectra.

The t-SNE results of real and generated samples by DCGAN of Zhetian 105 are shown in Figure 3. When 100 spectra were generated for each class, the generated samples exhibit a high degree of overlap with the real samples within the principal aggregation regions of each class. Classes 0 and 4 form relatively independent and compact clusters, while class 1 is predominantly distributed in the lower region; Classes 2 and 3 show partial overlap in certain local regions. The generated samples are distributed predominantly along the distribution trajectory of the real samples, without forming isolated clusters that deviate significantly from the real data, indicating that the DCGAN is capable of effectively learning the principal distribution characteristics of the real spectra of Zhetian 105. When the number of generated samples for each class is increased to 200, the coverage density of the generated samples on the real data manifold improves substantially. Classes 0 and 4 maintain well-defined inter-class boundaries, and the local structures of Classes 1, 2, and 3 are more comprehensively covered; however, a certain degree of overlap persists between Classes 2 and 3. The overall distribution pattern does not exhibit noticeable deviation, suggesting that increasing the number of generated samples enhances sample coverage while preserving the original class structure. When 300 samples are generated for each class, the generated samples provide relatively thorough coverage of the multiple local sub-clusters and continuous distribution regions formed by the real samples. Classes 0 and 4 retain strong inter-class separability, and although local intersections exist among Classes 1, 2, and 3 in adjacent regions, the generated samples remain generally consistent with the real samples of their corresponding classes. These results demonstrate that the DCGAN maintains favorable distribution fitting capability and a certain degree of sample diversity even at a larger generation scale.

**Figure 4:**
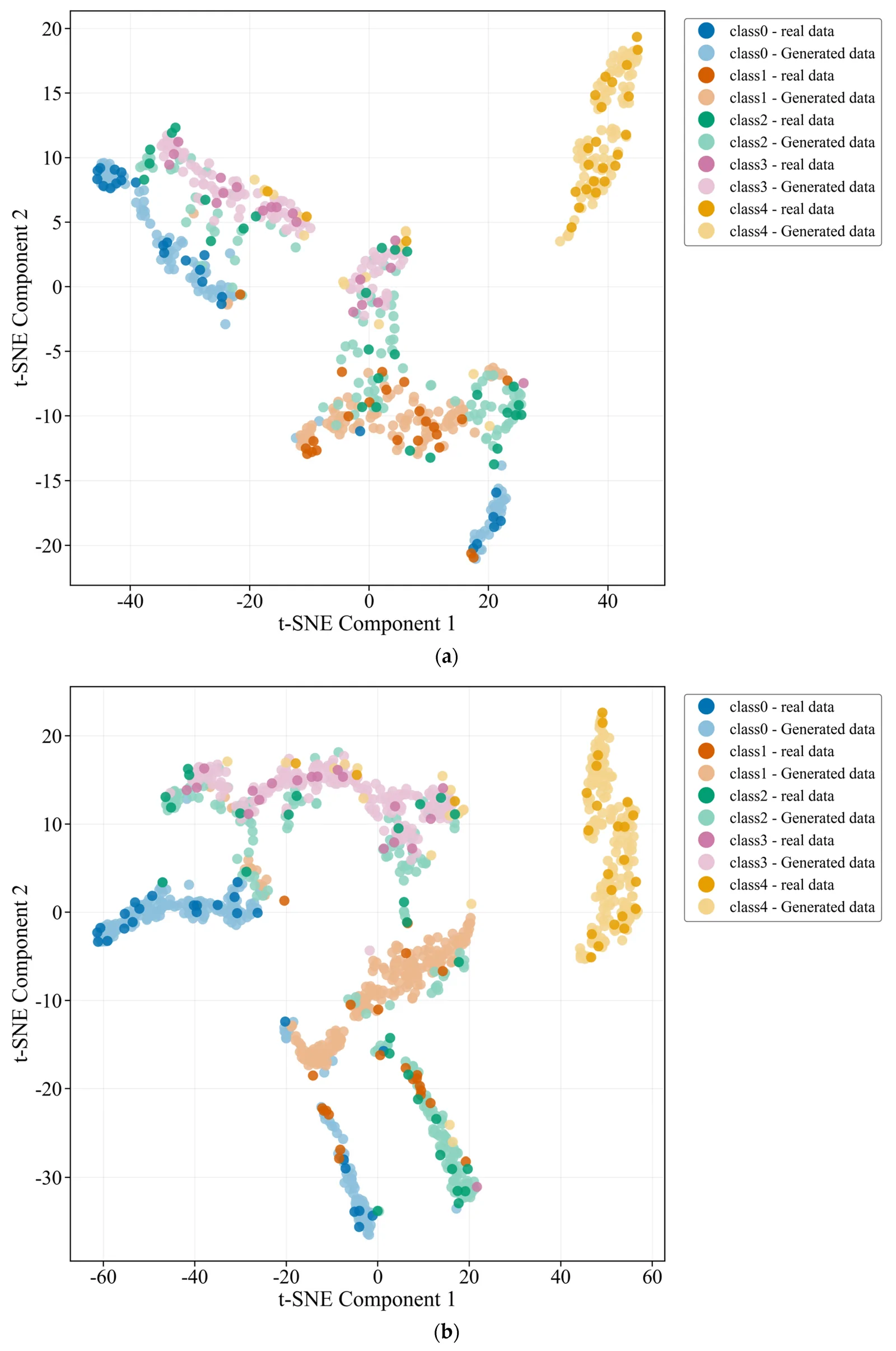

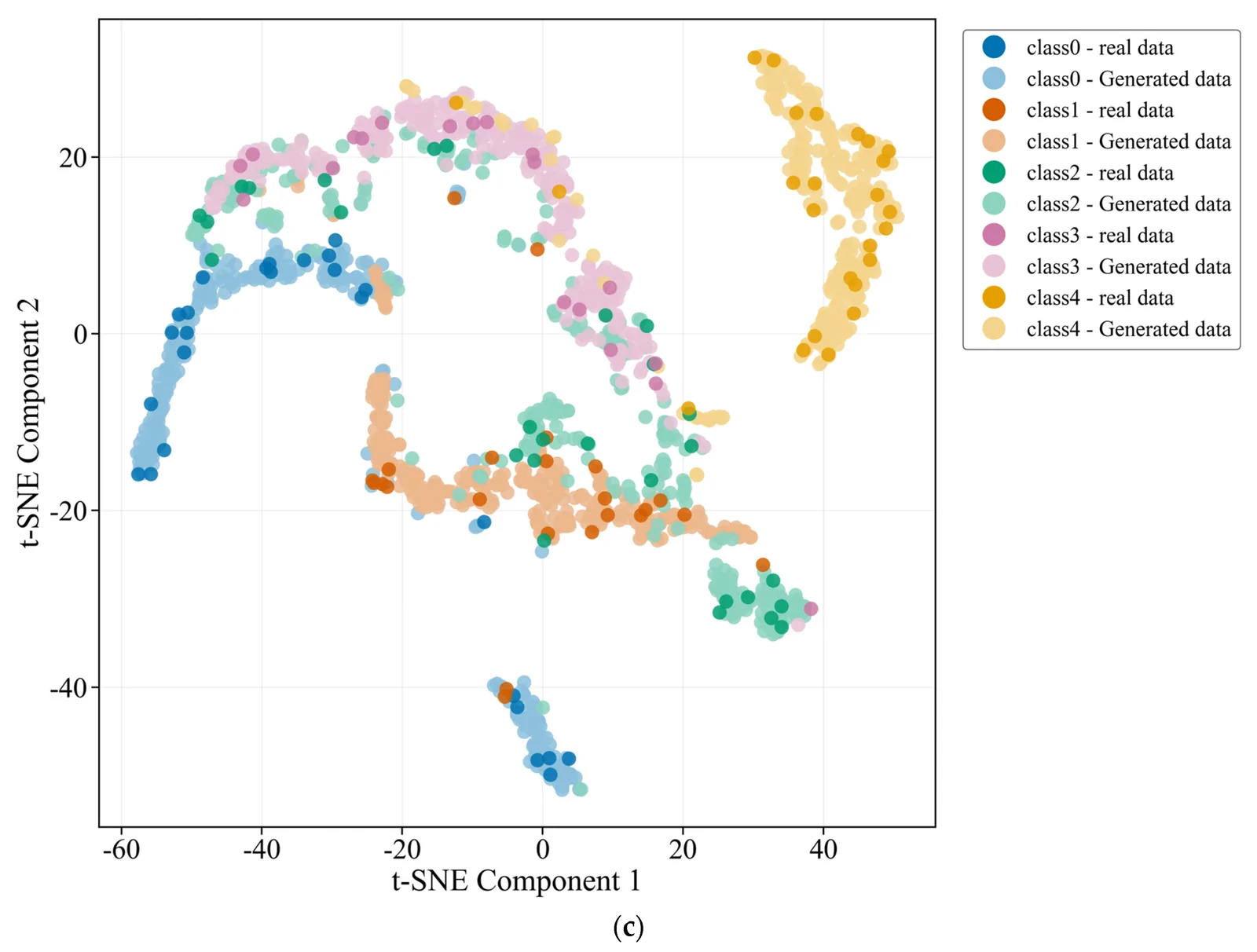
t-SNE visualizations comparing the generated data with the real data for the melon variety Zhetian 105 under CDCGAN: (**a**) 100 generated spectra; (**b**) 200 generated spectra; (**c**) 300 generated spectra.

The t-SNE results of real and generated samples by CDCGAN of Zhetian 105 are shown in Figure 4. For each class, 100 spectra were generated using the CDCGAN. The generated samples exhibit a high degree of overlap with the real samples within each class cluster and are continuously distributed along the local manifold of the real data. Classes 0 and 4 are well separated from the remaining classes, while class 1 is predominantly located in the lower region; classes 2 and 3 show partial overlap in certain transitional regions. No significant distribution drift is observed overall, indicating that the class-conditional information can effectively guide the CDCGAN to generate Zhetian 105 spectral samples that are consistent with the characteristics of the target classes. When the number of generated samples for each class is increased to 200, the coverage of the generated samples over the distribution trajectory of the real samples becomes more continuous across all classes. Classes 0, 1, and 4 maintain well-defined clustering structures. Although classes 2 and 3 are adjacent or overlapping in local regions, the generated samples remain predominantly distributed in the vicinity of their corresponding real samples. These results demonstrate that the CDCGAN is capable of preserving both the class structure and the intra-class distribution characteristics while augmenting the sample size. When the generation scale is further increased to 300 samples for each class, the generated samples form a relatively dense and continuous coverage within the real distribution region of each class. Classes 0 and 4 exhibit clearly independent distributions, and multiple local substructures of classes 1, 2, and 3 are well reproduced, with only minor overlap observed at the boundaries between adjacent classes. The overall topological structure remains stable, indicating that the CDCGAN possesses favorable generative stability, class consistency, and distributional coverage capability.

**Figure 5:**
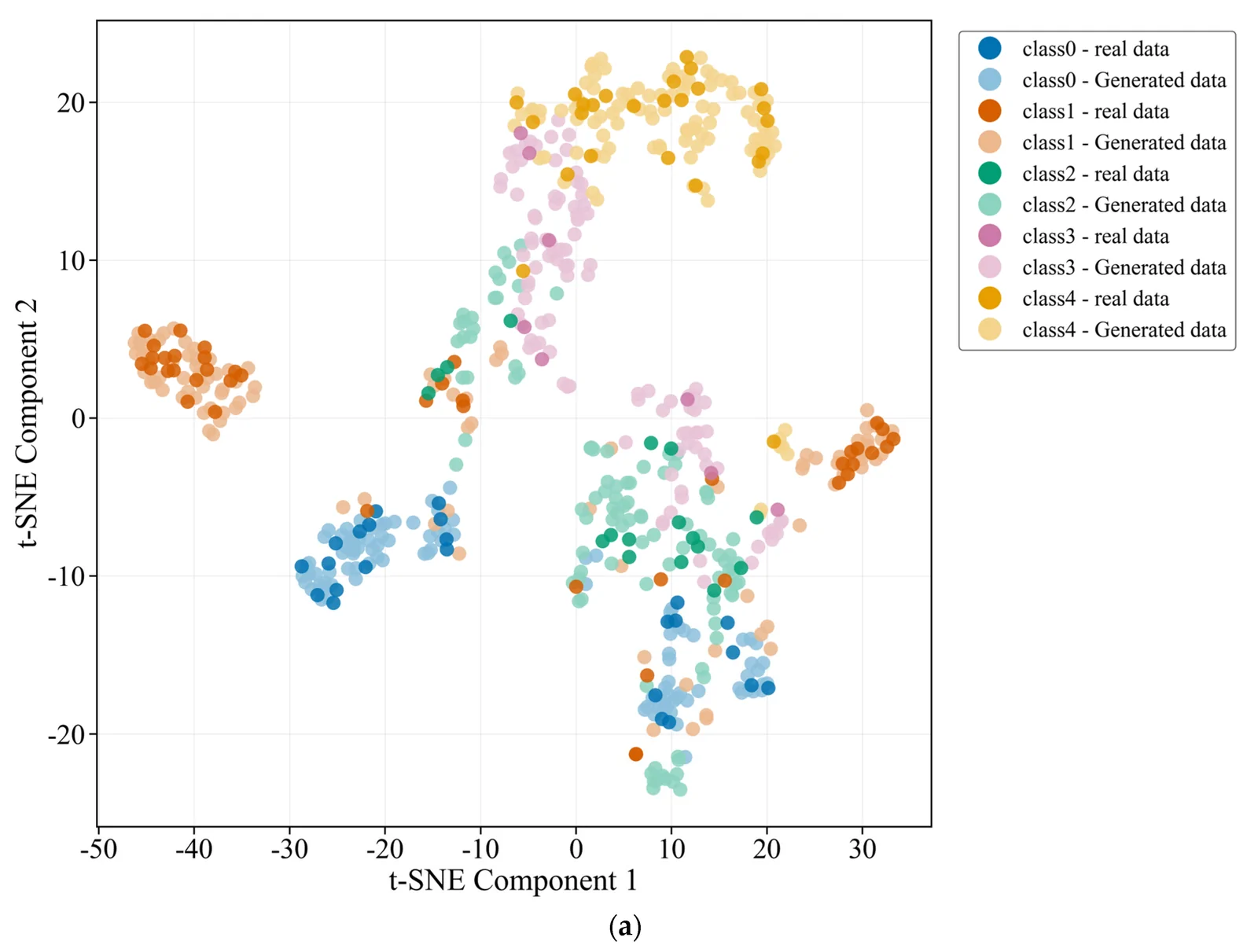

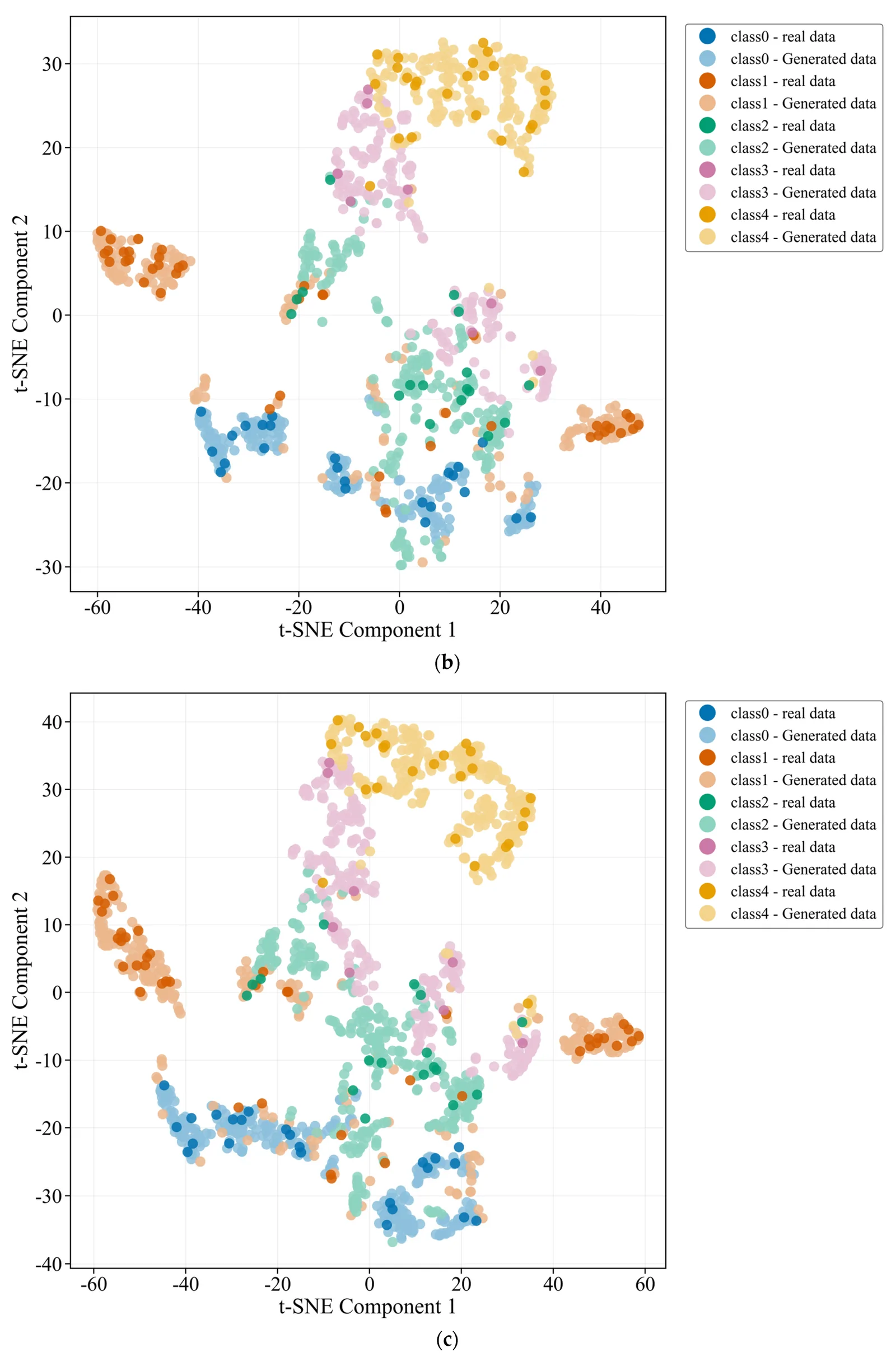
t-SNE visualizations comparing the generated data with the real data for the melon variety Zhetian 501 under DCGAN: (**a**) 100 generated spectra; (**b**) 200 generated spectra; (**c**) 300 generated spectra.

The t-SNE results of real and generated samples by DCGAN of Zhetian 501 are shown in Figure 5. For each class, 100 spectra were generated using the DCGAN. The generated samples are predominantly distributed in the vicinity of the real samples across all classes and are capable of reproducing multiple local clustering structures present in the real data. Classes 0, 1, and 4 exhibit relatively distinct distribution regions, whereas classes 2 and 3 show considerable overlap in the central region, with a small number of scattered points also observed. These results indicate that the DCGAN is able to learn the principal distribution characteristics of different classes, although certain limitations remain in discriminating the boundaries between similar classes. When the number of generated samples for each class is increased to 200, the coverage of the generated samples over the local sub-clusters of the real data becomes more comprehensive, while classes 0, 1, and 4 continue to maintain favorable inter-class separation. Classes 2 and 3 exhibit mixed distributions in certain regions, reflecting the inherent similarity in the spectral characteristics of these two classes. No large-scale deviation of the generated samples from the real distribution is observed, indicating that the overall distributional consistency remains satisfactory after increasing the number of generated samples. As the number of generated samples for each class is further increased to 300, the distribution density and coverage of the generated points in the two-dimensional space are further enhanced, and the generated samples are distributed predominantly along the manifolds formed by the real samples of each class. The clustering contours of classes 0, 1, and 4 remain relatively well defined, while local overlap and a small number of scattered points persist in the central region of classes 2 and 3. Overall, the DCGAN is capable of preserving the principal class structure of the Zhetian 501 dataset while generating samples with a certain degree of diversity.

**Figure 6:**
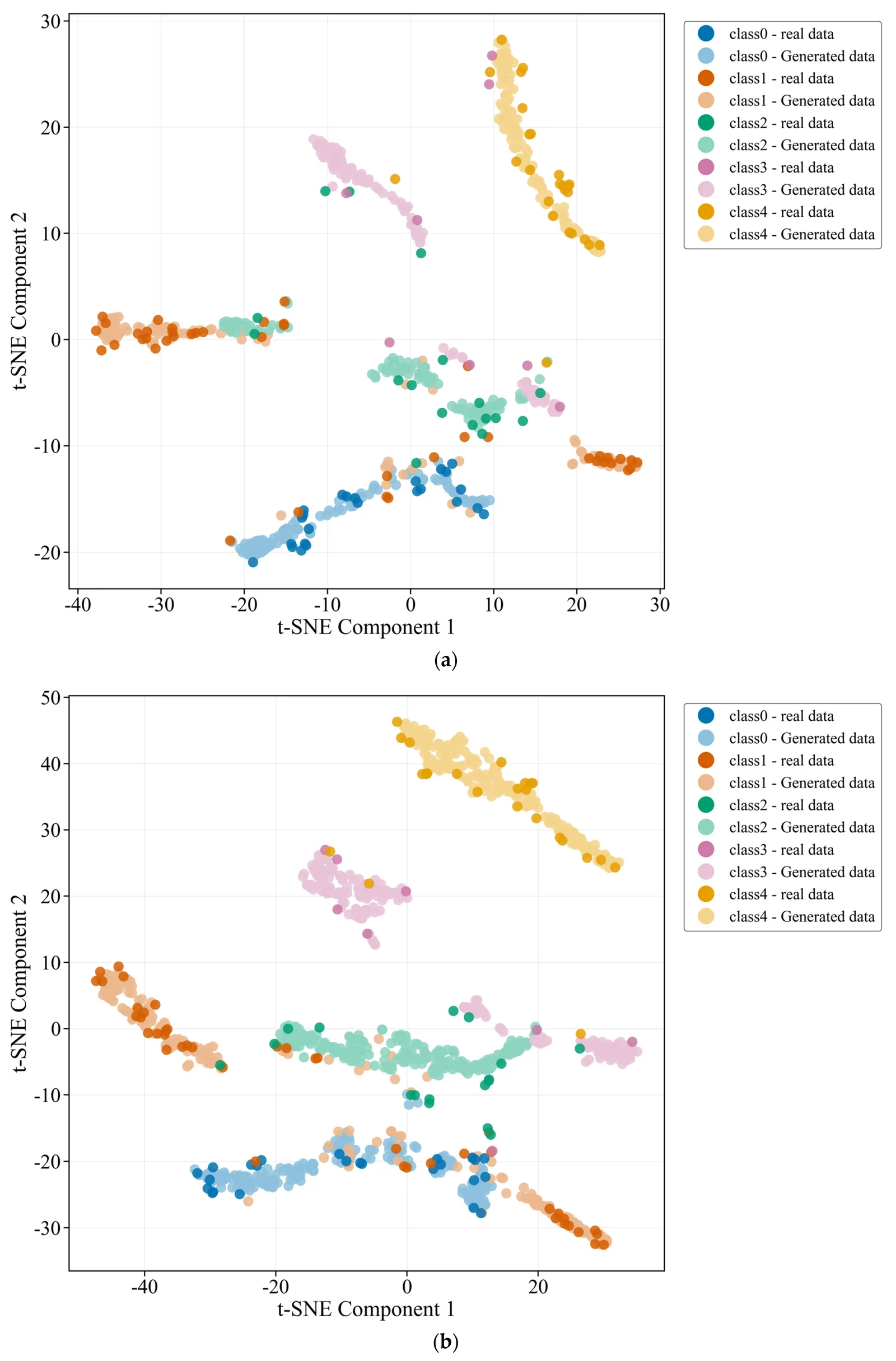

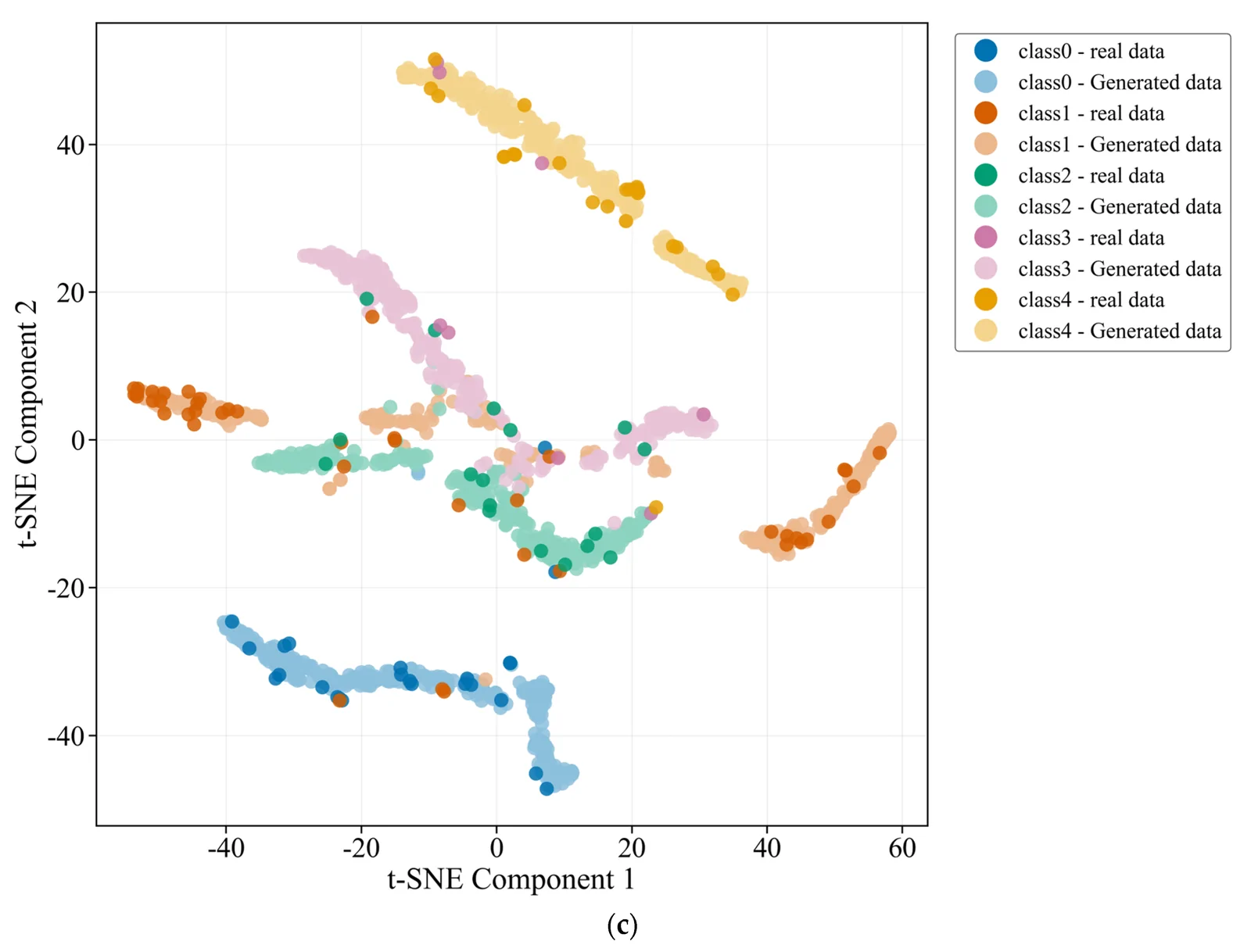
t-SNE visualizations comparing the generated data with the real data for the melon variety Zhetian 501 under CDCGAN: (**a**) 100 generated spectra; (**b**) 200 generated spectra; (**c**) 300 generated spectra.

The t-SNE results of real and generated samples by CDCGAN of Zhetian 501 are shown in Figure 6. For each class, 100 spectra were generated using the CDCGAN. The generated samples exhibit a high degree of consistency with the real samples along the local trajectories of each class. Classes 0, 1, and 4 form mutually separated band-like or cluster-like structures, while classes 2 and 3 are also predominantly distributed within their respective real sample regions, with overlap occurring only at a limited number of boundary locations. Compared with the unconditional generation results, the class structure in the figure is more regular, indicating that the class-conditional constraint contributes to improved class consistency of the generated samples. When the number of generated samples for each class is increased to 200, the generated samples reproduce the continuous manifold and multiple local sub-clusters of the real data with relatively complete fidelity. The distribution boundaries of classes 0, 1, and 4 remain well defined, and the majority of the generated samples for classes 2 and 3 closely coincide with their corresponding real samples, with only minor local intersections observed. These results demonstrate that the CDCGAN is capable of maintaining favorable distribution fitting accuracy while increasing the number of generated samples. When the number of generated samples for each class is further increased to 300, the generated samples are densely distributed along the band-like manifolds formed by the real samples, and the principal structures of all five classes are well preserved. The generated samples across all classes exhibit a high degree of overlap with the real samples, and no anomalous clusters that are clearly separated from the real distribution are observed, with the separability of Classes 0, 1, and 4 being particularly distinct. These results indicate that the CDCGAN possesses favorable generative stability, class preservation capability, and sample diversity on the Zhetian 501 dataset.

#### 2.3.3. Quality Assessment of Generated Spectra Using Classification Models

To further evaluate the quality of the generated spectra, this study adopted two strategies. In Strategy I, a classification model was built using generated spectra to predict the real test samples. In Strategy II, a classification model was built using the generated data with real training samples to predict the real test samples. To enable a more comprehensive evaluation of the generated data quality, LR, SVC, XGBoost, and CNN models were constructed, thereby avoiding results that might be specific to a particular model or dataset.

Table 3 presents the prediction results of the classification models constructed with varying numbers of samples generated by DCGAN and CDCGAN for Zhetian 105, evaluated on the real-sample test set. For DCGAN, when the number of generated samples per class was 100, the SVC model achieved the highest classification accuracy, followed by the CNN, XGBoost, and LR models. When the number of generated samples per class was 200, the SVC model yielded the best performance, followed by CNN, XGBoost, and LR. When the number of generated samples per class was 300, the SVC model again achieved the highest classification accuracy, followed by XGBoost, CNN, and LR. Compared with the baseline results obtained using the real training set shown in Table 2, when classifiers were trained exclusively on DCGAN-generated samples, the test accuracy of most models improved across all three generation quantities. These results demonstrate that DCGAN-generated samples can improve the overall classification performance for Zhetian 105. However, across different generation quantities, the prediction accuracy on the real-sample test set did not consistently increase with the number of generated samples per class, and the performance varied among different models.

For CDCGAN, when the number of generated samples per class was 100, the SVC model achieved the highest classification accuracy, followed by the CNN, XGBoost, and LR models. When the number of generated samples per class was 200, the SVC model yielded the best performance, followed by CNN, XGBoost, and LR. When the number of generated samples per class was 300, the SVC model again achieved the highest classification accuracy, followed by XGBoost, CNN, and LR. Compared with the baseline results obtained using the real training set shown in Table 2, when classifiers were trained exclusively on CDCGAN-generated samples, the test accuracy of all models improved across all three generation quantities. These results indicate that CDCGAN-generated samples can generally enhance the classification performance for Zhetian 105. However, across different generation quantities, the prediction accuracy on the real-sample test set did not consistently increase with the number of generated samples per class, and the performance varied among different models.

Table 4 presents the prediction results of the classification models constructed with the real training samples augmented by varying numbers of samples generated by DCGAN and CDCGAN for Zhetian 105, evaluated on the real-sample test set. For DCGAN, when the number of generated samples per class was 100, the SVC model achieved the highest classification accuracy, followed by the CNN, XGBoost, and LR models. When the number of generated samples per class was 200, the SVC and CNN models yielded the best performance, followed by XGBoost and LR. When the number of generated samples per class was 300, the SVC model again achieved the highest classification accuracy, followed by XGBoost, CNN, and LR. Compared with the baseline results obtained using the real training set shown in Table 2, the test accuracy of all four classifiers improved after augmenting the real training set with DCGAN-generated samples. These results indicate that augmenting the real training samples with DCGAN-generated samples can generally enhance the classification performance for Zhetian 105. However, across different generation quantities, the prediction accuracy on the real-sample test set did not consistently increase with the number of generated samples per class.

For CDCGAN, when the number of generated samples per class was 100, the SVC and CNN models achieved the highest classification accuracy, followed by the XGBoost and LR models. When the number of generated samples per class was 200, the SVC model yielded the best performance, followed by XGBoost, CNN, and LR. When the number of generated samples per class was 300, the CNN model achieved the highest classification accuracy, followed by SVC, XGBoost, and LR. Compared with the baseline results obtained using the real training set shown in Table 2, the test accuracy of all four classifiers improved across all three generation quantities after augmenting the real training set with CDCGAN-generated samples. These results demonstrate that augmenting the real training samples with CDCGAN-generated samples can generally enhance the classification performance for Zhetian 105. However, across different generation quantities, the prediction accuracy on the real-sample test set did not consistently increase with the number of generated samples per class.

Table 5 presents the prediction results of the classification models constructed with varying number of samples generated by DCGAN and CDCGAN for Zhetian 501, evaluated on the real-sample test set. For DCGAN, when the number of generated samples per class was 100, the SVC model achieved the highest classification accuracy, followed by the CNN, XGBoost, and LR models. When the number of generated samples per class was 200, the CNN model yielded the best performance, followed by SVC, LR, and XGBoost. When the number of generated samples per class was 300, the CNN model achieved the highest classification accuracy, followed by SVC and LR, with XGBoost ranking last. Compared with the baseline results obtained using the real training set shown in Table 2, when classifiers were trained exclusively on DCGAN-generated samples, the test accuracy of all models improved across all three generation quantities. These results indicate that DCGAN-generated samples can improve the overall classification performance for Zhetian 501. However, across different generation quantities, the prediction accuracy on the real-sample test set did not consistently increase with the number of generated samples per class.

For CDCGAN, when the number of generated samples per class was 100, the SVC model achieved the highest classification accuracy, followed by the CNN, XGBoost, and LR models with comparable performance. When the number of generated samples per class was 200, the CNN model yielded the best performance, followed by SVC, LR, and XGBoost. When the number of generated samples per class was 300, the SVC model achieved the highest classification accuracy, followed by CNN, LR, and XGBoost. Compared with the baseline results obtained using the real training set shown in Table 2, when classifiers were trained exclusively on CDCGAN-generated samples, the test accuracy of the majority of models improved across all three generation quantities. These results demonstrate that CDCGAN-generated samples can improve the overall classification performance for Zhetian 501. However, across different generation quantities, the prediction accuracy on the real-sample test set did not consistently increase with the number of generated samples per class.

Table 6 presents the prediction results of the discriminant analysis models constructed with the real training samples augmented by varying number of samples generated by DCGAN and CDCGAN for Zhetian 501, evaluated on the real-sample test set. For DCGAN, when the number of generated samples per class was 100, the CNN model achieved the highest classification accuracy, followed by the SVC, XGBoost, and LR models. When the number of generated samples per class was 200, the CNN model yielded the best performance, followed by SVC, LR, and XGBoost. When the number of generated samples per class was 300, the CNN model again achieved the highest classification accuracy, followed by SVC, LR, and XGBoost. Compared with the baseline results obtained using the real training set shown in Table 2, the classification accuracy of all models on the test set improved after augmenting the real training set with DCGAN-generated samples. These results indicate that augmenting the real training samples with DCGAN-generated samples can generally enhance the classification performance for Zhetian 501. However, across different generation quantities, the prediction accuracy on the real-sample test set did not consistently increase with the number of generated samples per class.

For CDCGAN, when the number of generated samples per class was 100, the CNN model achieved the highest classification accuracy, followed by the SVC, XGBoost, and LR models. When the number of generated samples per class was 200, the CNN model yielded the best performance, followed by SVC, LR, and XGBoost. When the number of generated samples per class was 300, the SVC model achieved the highest classification accuracy, followed by CNN, LR, and XGBoost. Compared with the baseline results obtained using the real training set shown in Table 2, the classification accuracy of all models on the test set improved after augmenting the real training set with CDCGAN-generated samples. These results demonstrate that augmenting the real training samples with CDCGAN-generated samples can generally enhance the classification performance for Zhetian 501. However, across different generation quantities, the prediction accuracy on the real-sample test set did not consistently increase with the number of generated samples per class.

As shown in Table 3, Table 4, Table 5 and Table 6, it could be found that the classification accuracy of the training set of SVC, XGBoost, and CNN was quite high, and the classification accuracy of the training set of SVC can reach 100%. These results indicated that the samples of each class generated by DCGAN and CDCGAN showed high separability with the other classes.

#### 2.3.4. Class-Wise Analysis of Real Test Samples of SVC Models

In addition to the overall performance analysis, class-wise analysis of the real test samples was also conducted. Since there were a lot of datasets and models, it is quite difficult to analyze all the models. Since SVC and CNN models obtained relatively better performance, the test results of all SVC models were analyzed. The class-wise prediction results of the real test samples by all SVC models of Zhetian 105 and Zhetian 501 are shown in Tables S3 and S4.

As for Zhetian 105, it could be noted that the prediction accuracy varied from different classes. The prediction results obtained from the models using different numbers of generated samples by DCGAN showed that there were decreases in class 1 and class 3 compared with the results obtained from the model using real training samples. While for the models using different number of generated samples by CDCGAN, the prediction re-sults of class 3 decreased. When the real samples with generated samples as the training set, the prediction results of corresponding classes still decreased, and the decrease in the pre-diction results was smaller. Under all the conditions, the prediction results of class 0 (healthy samples) highly increased. As for Zhetian 501, the decrease in prediction results mainly occurred on class 4, and the decrease was not large. As for class 3, the prediction accuracy was 0% from the model established using real training samples. With the generated data, the prediction accuracy of class 3 improved significantly. Although there were only eight real training samples for class 3, the results indicated the potential and feasibility of the DCGAN and CDCGAN under limited samples. However, more real samples were needed for better data generation performance.

Although the overall performances increased with generated samples, the prediction performance of certain class decreased. Thus, it was important to further investigate this issue.

## 3. Discussion

In this study, we employed three different generative adversarial networks (CGAN, DCGAN, and CDCGAN) to generate leaf reflectance spectra for two melon varieties under different levels of powdery mildew stress. The results demonstrate the feasibility of using GANs for this task. Various improved GAN models have been proposed in the literature [23,32,33,35]; the three models used here were chosen primarily because they have been validated to a certain extent for spectral data generation [23,36]. A limitation observed with CGAN is that, even after extensive hyperparameter tuning, the generated spectra still contain noticeable noise, and the reasons for this phenomenon should be further investigated.

Because spectral features are closely linked to the internal physicochemical components of the studied objects, differences in generated spectra may, to some extent, reflect differences in material composition. It is possible for generated spectra to display consistent overall trends yet differ in specific features, resulting in spectra that do not meet the intended requirements. Generating high-quality spectral data is therefore critical for spectral data analysis. In this study, DCGAN generated spectra based on the spectral features of samples belonging to the same class, whereas CGAN and CDCGAN both incorporated class-conditional information. Given the particular nature of spectral data, researchers have widely adopted diverse strategies to obtain high-quality generated data [23,32,33].

Currently, the evaluation of generated spectral data primarily relies on visualizing the spectral curves, t-SNE distributions, and incorporating the generated data into model construction [23,32,33]. In this study, SAM, WD, and RMSE were also used, and spectra generated by DCGAN and CDCGAN showed low SAM, WD, and RMSE values, indicating good quality of the generated spectra.

In this study, different numbers of samples were generated for each class (100, 200, and 300 for each class). However, the classification models using different numbers of generated samples and the classification models using the combination of real training samples and generated samples showed improvements on the prediction of the real test samples. However, no specific patterns of the improvement with different numbers of generated samples. The overall results illustrated the feasibility of generating the samples of each disease severity. However, the phenomenon that generating more spectra would not obtain better performance indicated that diffusibility of the generated data was limited. The sample generation based on limited real training samples may also result in insufficient representativeness.

Although the overall classification performance increased with generated spectra in most cases, the performance of single class varied. The prediction performance of some classes decreased; this phenomenon could be attributed to the small number of real training samples and the insufficient representativeness of the generated spectra. More work should be done in the future studies.

In this study, no specific patterns were observed with different numbers of generated samples. However, only one sing-run of data generation was conducted in this study, which limited the generalization ability of the proposed methods. In future studies, it will be better to conduct more runs of data generation to more comprehensively evaluate the performance of DCGAN and CDCGAN. Moreover, in this study, the plants from which the leaves were collected, the sample acquisition dates, and the sampling batches were not considered during the data analysis. The samples were collected for different classes, and the reported performance may be optimistic for fully independent plants, acquisition dates, and batches. In future studies, the sampling plants, dates, and batches should be considered for better performance and generalization ability.

In addition, the reliability of generated spectral data should be further considered from the perspective of domain knowledge. In this study, disease severity labels were determined based on expert assessment and disease grading criteria. However, the current framework still mainly relies on data-driven learning and has not directly embedded plant pathological knowledge, disease progression rules, characteristic wavelength information, or expert-defined severity constraints into the generative models. Future studies may further develop knowledge-guided or expert-informed spectral generation frameworks to improve the biological plausibility and practical reliability of generated samples.

In future work, it is necessary to increase the number of real samples to improve the coverage and representativeness of real sample features, and to analyze and predict unknown real samples to further evaluate the quality of data generated from a limited number of real samples. The external validation of the generated data based on unknown real samples should also be further investigated. Moreover, the improved GAN models can also be developed to generate data with diversity and diffusibility from small numbers of real samples, which can be directly used for unknown real samples with good performance. Meanwhile, beyond assessing the spectral shape and trends, it is also necessary to generate and analyze specific characteristic features to enhance the quality of the generated data. Future research should also consider and propose new strategies for generating data and evaluating the quality of generated samples.

## 4. Materials and Methods

### 4.1. Sample Preparation

**Figure 7:**
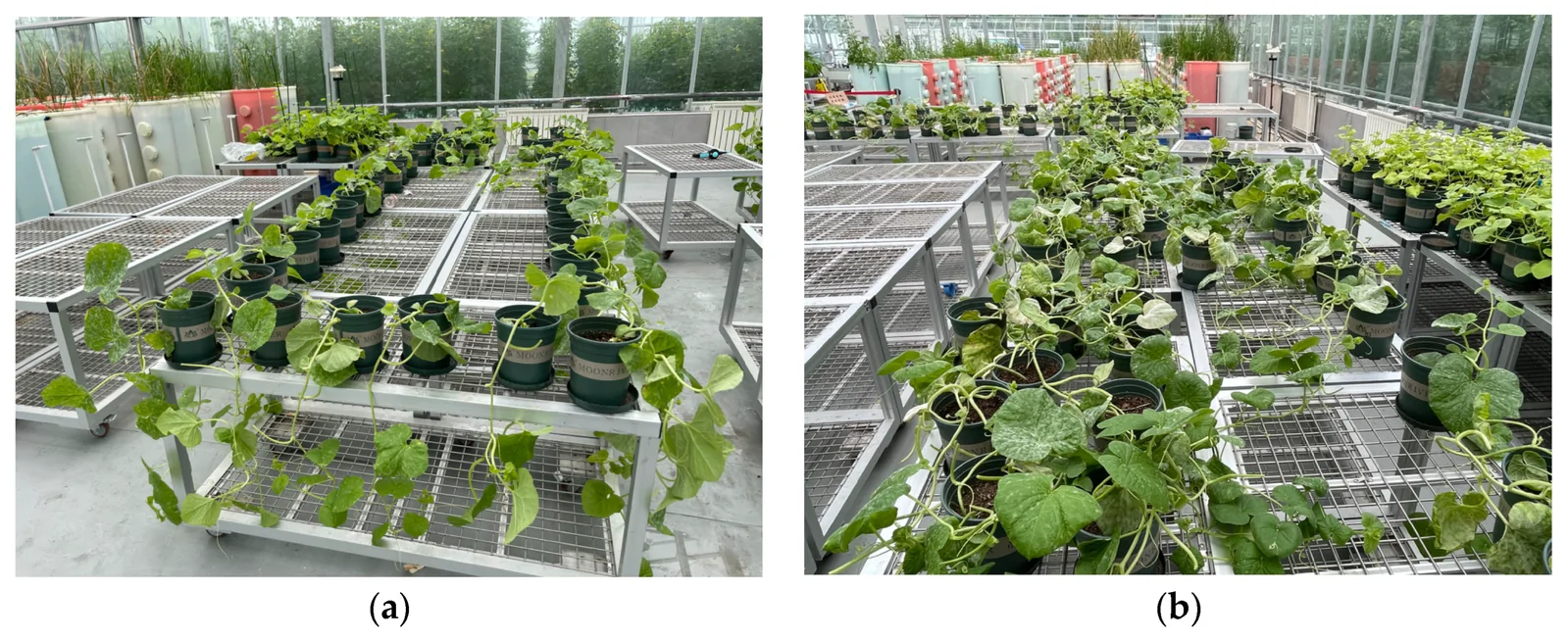
Melon plants grown in a greenhouse after inoculation with powdery mildew: (**a**) Zhetian 105; (**b**) Zhetian 501.

This study focused on melon leaves infected with powdery mildew. Two melon varieties with contrasting resistance, Zhetian 105 and Zhetian 501, were selected and cultivated in a substrate within an artificial climate greenhouse (shown in Figure 7). For each variety, 40 plants were used, with 30 for disease inoculation and 10 for healthy control. The healthy and inoculated plants were managed separately in different rooms with the same water and fertilizer treatment.

When the seedlings reached the three-leaf stage with one central bud, they were uniformly inoculated with powdery mildew pathogen (*Podosphaera xanthii*). Powdery mildew isolates were obtained from naturally infected muskmelon leaves at the early stage of disease onset in a field at the Zhejiang Academy of Agricultural Sciences. Conidia were collected from individual lesions using a sterile, moistened brush and suspended in 1 mL of distilled water in separate microcentrifuge tubes. Each suspension was subjected to microscopic examination and DNA sequencing. Following morphological and molecular confirmation of pathogen identity, suspensions derived from the same isolate were pooled and adjusted to a final concentration of 10^5^ conidia/mL. Genomic DNA was extracted from the fungal samples. The full-length internal transcribed spacer (ITS) region of the ribosomal DNA (rDNA) was amplified using the universal primers ITS1 and ITS4, and subsequently sequenced. The resulting ITS-rDNA sequences were aligned against the NCBI GenBank database to determine the taxonomic classification of the isolate.

The cultivation substrate was sterilized by steam at 100 °C for 10 min and subsequently transferred into 1-gallon pots. Muskmelon plants were grown in an isolated growth chamber at the Agricultural Science and Innovation Experimental Center, Zhejiang University, under a 12 h light/12 h dark photoperiod, with day/night temperatures set at 30 °C/20 °C, respectively, until the five-leaf-one-heart stage was reached. Inoculation was performed after 16:00 by uniformly spraying the conidial suspension onto the leaves of the isolated plants until runoff, without causing dripping. Relative humidity (RH) was maintained above 85% for the first 12 h post-inoculation (hpi). Thereafter, the plants were cultivated under a day/night temperature regime of 28 °C/20 °C, with RH maintained above 85% during the night and uncontrolled during the day. Normal management was resumed 12 h post-inoculation. Upon disease symptom development, conidial suspensions were randomly collected from five independent lesions for microscopic examination and sequencing to verify the pathogen.

From 12 April to 2 June after inoculation, the symptoms of disease appeared at different times, the leaf samples at different disease severity were continuously collected at different dates, and hyperspectral image data were then acquired using a hyperspectral imaging system on the same day. The imaging system settings kept the same during the experiments. Figure 7a shows a Zhetian 105 melon plants after powdery mildew inoculation, and Figure 7b shows a Zhetian 501 melon plants after inoculation.

The leaves were detached, placed in an ice box for simultaneous dark adaptation and transferred to the laboratory for hyperspectral image data acquisition. Through systematic identification and grading of the powdery mildew-infected leaves by multiple plant protection experts, a total of 475 hyperspectral images representing different levels of disease severity were finally selected to construct a dataset of melon leaf samples.

Since no standard for grading powdery mildew severity on melon leaves has been found in the literature, this study extensively reviewed relevant materials and, following the joint recommendations of three experts from the Institute of Plant Protection and Microbiology at the Zhejiang Academy of Agricultural Sciences and professors of plant pathology from the Institute of Biotechnology at Zhejiang University, referred to grading standards for disease severity in other plants.

Accordingly, the severity of powdery mildew on melon leaves was divided into five levels, with the detailed grading criteria presented in Table S5 (in Supplementary Materials), representative leaves of the Zhetian 105 and Zhetian 501 melon cultivars at different disease severity levels are presented in Figures S1 and S2 (in Supplementary Materials), respectively. The severity grading was confirmed by all the three experts. During the grading, the three experts graded the disease severity level independently, the leaf with the same grade from all three experts was kept for further analysis, and the leaf with different grades from all three experts was excluded for analysis. After the grading, 475 leaves with the same grades from all the three experts were used for analysis. The final distribution of samples under different levels of disease stress for the two melon varieties is summarized in Table 7.

### 4.2. Hyperspectral Image Acquisition

The hyperspectral imaging system was composed of a hyperspectral imager (Imspector V10E, Specim, Spectral Imaging Ltd., Oulu, Finland), a dark chamber, a conveyor belt, a computer, and two halogen lamps, which provided stable illumination over the wavelength range of 350–2500 nm. The imager operated within a spectral range of 400–1000 nm, and the number of spectral bands was set to 512, with an image resolution of 512 × 473 pixels. The halogen lamps were positioned at a 45° downward angle and placed approximately 50 cm from the sample to ensure uniform illumination; once fixed, the light source positions remained unchanged throughout the experiment. Prior to each data acquisition session, the system was warmed up for 30 min. Subsequently, in the image acquisition software, the integration time was set to 65 ms, the conveyor belt speed was set to 7 mm/s, and the distance from the imager to the moving platform was set to 360 mm. A calibration panel was recorded before each acquisition, after which the leaves were sequentially placed onto the conveyor belt, and hyperspectral images of each sample were obtained by moving the belt.

The acquired hyperspectral images of melon leaves required radiometric correction. Since the leaves were sampled on different days, reference images of a white board and a black board were reacquired before each experiment. The white board was a polytetrafluoroethylene (Teflon) standard plate with near 100% reflectance, while the black board reference was obtained by covering the hyperspectral camera lens with a lens cap of near-zero reflectance. After the acquisition of hyperspectral images, the images were corrected using Equation (1), where *I_Ref_* is the corrected hyperspectral image, *I_Raw_* is the raw hyperspectral image, *I_D_* is the black board image, and *I_W_* is the white board image.


(1) $$I_{Ref}=\frac{I_{Raw}-I_{D}}{I_{W}-I_{D}}$$

### 4.3. Spectral Data Extraction

**Figure 8:**
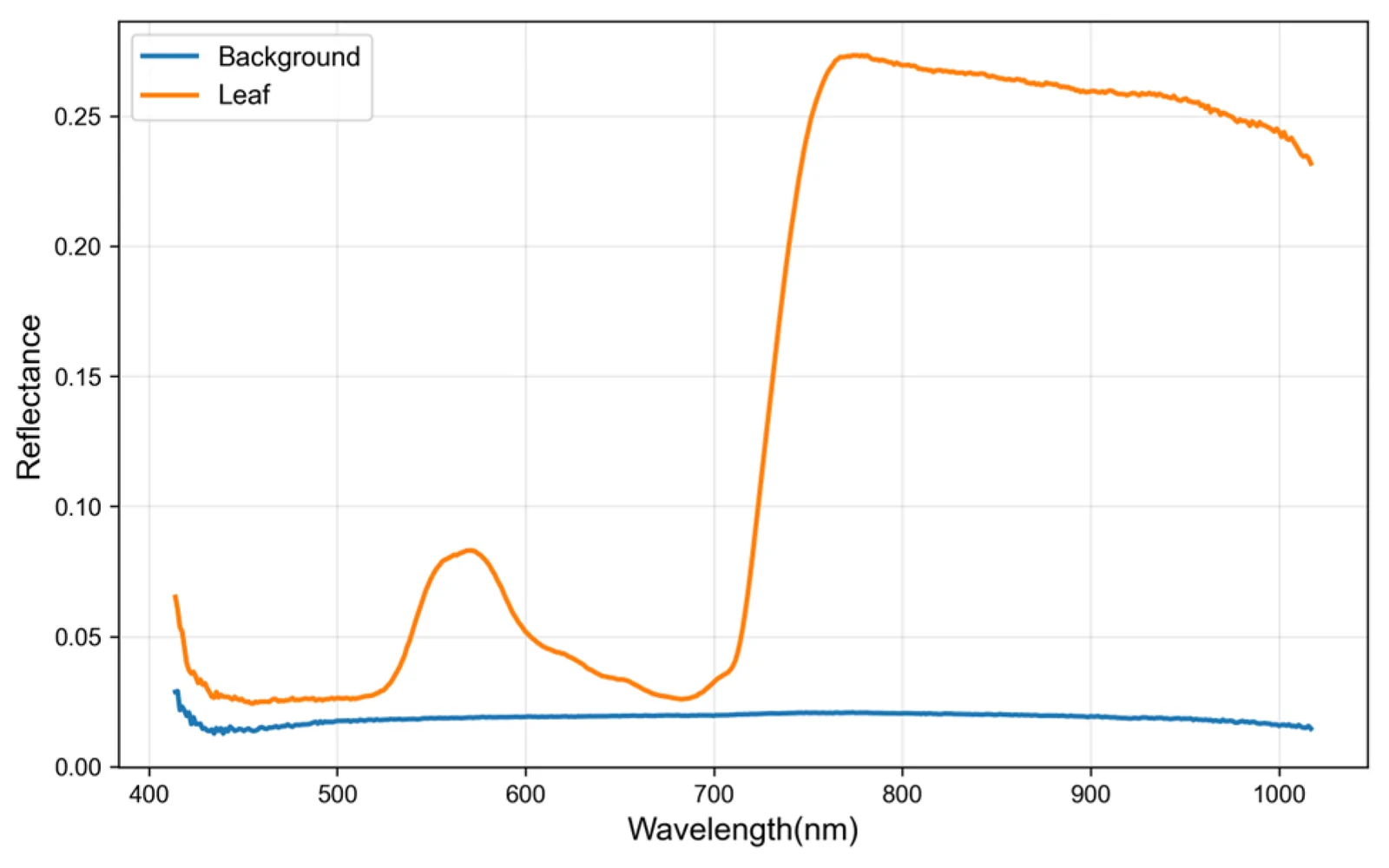
The reflectance spectral curves of the hyperspectral image background and leaf regions.

After correction, the hyperspectral image showed a clear difference in reflectance between the background and the leaves (as shown in Figure 8). Since the image mainly consisted of leaves and background, we applied binarization to the grayscale image at 800 nm to remove the background. In the binarized image, the background had a reflectance of 0, while the leaf area had a reflectance of 1. We then used the binarized image as a mask for each spectral band and extracted the average spectrum of the entire leaf region of interest (ROI). Due to noise from the hyperspectral imager response, we removed the noisy signals at both ends of the spectrum and retained the range of 475–987 nm (totaling 400 bands). To further reduce the impact of noise on the results, we preprocessed the spectral data using moving average smoothing with a smoothing window of 7 points.

### 4.4. Classification Model

Logistic regression (LR) is a widely used classification algorithm [37]. It maps the output of linear regression to the interval [0, 1] via the sigmoid function, thereby yielding class probabilities. The basic LR algorithm is originally designed for binary classification and can be extended to multi-class tasks using various strategies. In this study, the LR function from Scikit-learn was employed, with L2 regularization to mitigate overfitting (regularization strength C = 1.0). The liblinear optimizer was selected to accommodate high-dimensional spectral data and small sample scenarios. The multi-class strategy was One-vs-Rest (OVR), the maximum number of iterations was set to 20,000, and other parameters used their default values.

Support vector classification (SVC) is a widely used algorithm [38]. Originally designed for binary classification, SVC handles linearly separable data by maximizing the margin between two classes via an optimal hyperplane. For nonlinear data, kernel functions map samples into a high-dimensional space to achieve linear separability. SVC can be extended to multi-class tasks. In this study, the SVC function from Scikit-learn was employed, with 5-fold cross-validation for hyperparameter tuning to accommodate the nonlinear characteristics and class distribution of high-dimensional spectral data. The hyperparameter search space was set as follows: penalty parameter C = [0.01, 0.1, 1, 10, 100]; kernel coefficient gamma = [0.001, 0.01, 0.1, 1, 10, 100]; kernel functions were rbf and linear. The optimization objective was cross-validation accuracy.

eXtreme Gradient Boosting (XGBoost) is a widely used machine learning method for classification and regression [39]. XGBoost is a gradient boosting decision tree algorithm, where each tree is trained on the residuals of the previous tree, iteratively optimizing the loss function to reduce the residuals between true and predicted values. It accelerates training via parallel computing and reduces overfitting through tree pruning and regularization. In this study, we employed the Python XGBoost library for a five-class classification task with the following hyperparameters: multi-class log loss as the evaluation metric; max depth = 4; learning rate = 0.008; number of trees = 1200. To enhance generalization, we set subsample = 0.8, colsample_bytree = 0.8, and min_child_weight = 12. L1 regularization (alpha = 1) and L2 regularization (reg_lambda = 1) were applied to prevent overfitting.

The Convolutional Neural Network (CNN) is the most fundamental and widely used deep learning architecture, extensively applied in spectral data analysis [40]. A CNN consists of convolutional layers for local feature extraction, pooling layers for dimensionality reduction and computational efficiency, and fully connected layers for feature integration and classification/regression. Its flexible structure allows adjustments in layer number, parameters, and the incorporation of normalization, dropout, etc. Convolutional layers can be 1D, 2D, or 3D, enabling processing of corresponding data types. Attention mechanisms are common deep learning techniques that focus on task-relevant features for more efficient data processing. Among various attention methods, Efficient Channel Attention (ECA) is an effective lightweight module. In this study, ECA was adopted as the primary attention mechanism in the CNN model.

**Figure 9:**
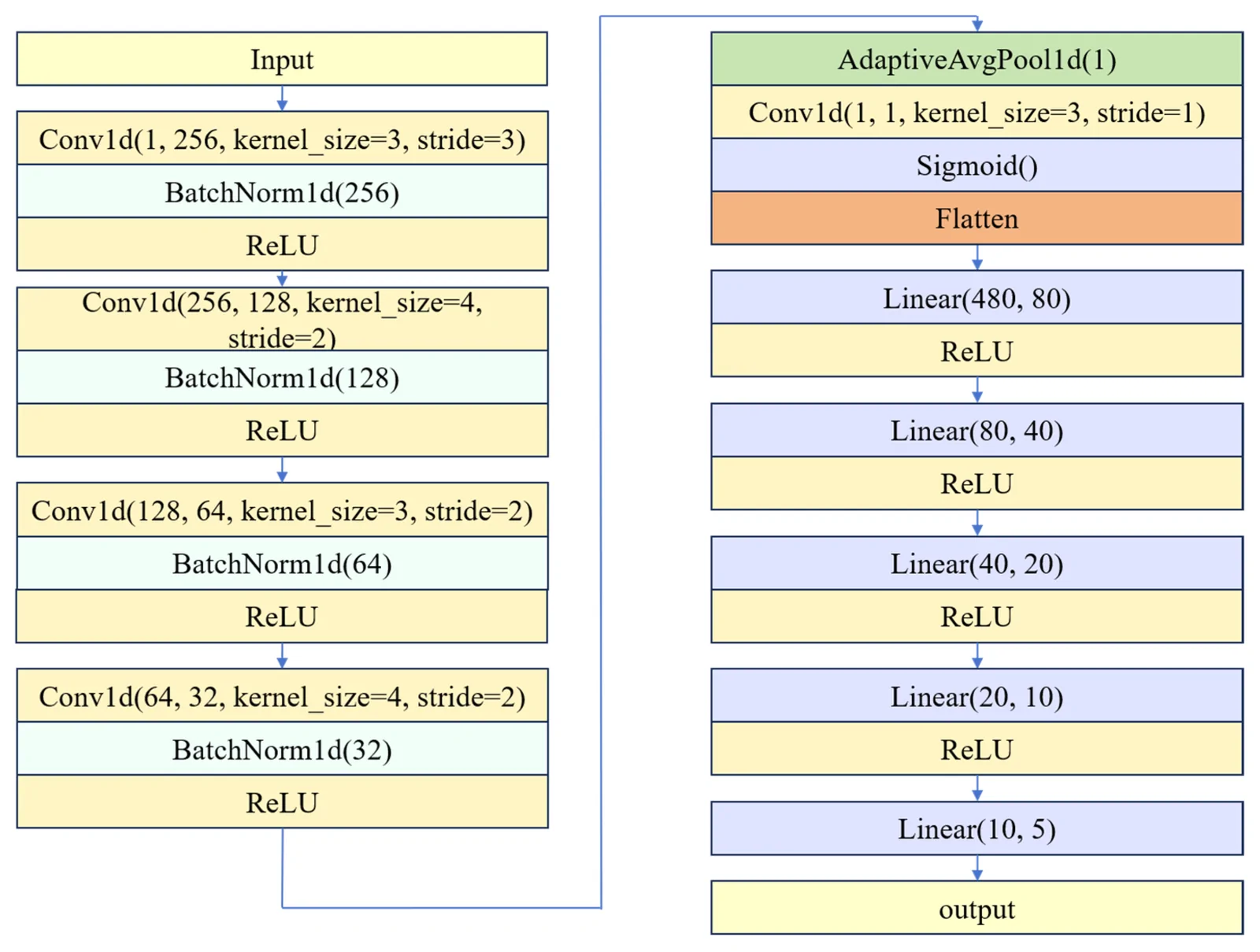
Framework of CNN models for all datasets of Zhetian105 and Zhetian 501.

The CNN architecture—specifically the number of convolutional and fully connected layers—was determined via trial and error. The same CNN architecture was used for all the datasets. Figure 9 illustrates the CNN framework for all the datasets. During the CNN training, the default hyperparameters were as follows: EPOCH = 500. The optimizer was set as Adam, and the loss function was set as CrossEntropyLoss(). For CNN model establishment, a three-fold cross-validation was used to obtain the optimal combination of BATCH_SIZE and learning rate. The searching lists of BATCH_SIZE and learning rate were set as [0.001, 0.005, 0,1] and [4, 8, 16, 32, 64]. The hyperparameters determined by three-fold cross-validation of CNN models are presented in Table S6.

### 4.5. Data Generation Methods Using Different Generative Adversarial Networks

The Generative Adversarial Network (GAN) is a deep learning framework that realizes data generation through adversarial learning between a generator and a discriminator [41,42]. The generator produces fake samples, while the discriminator judges whether the generated samples are real or fake. To meet various generation requirements, numerous GAN variants have been developed.

The Conditional Generative Adversarial Network (CGAN) is an important extension of GAN [22,36]. In standard GAN-based data generation, only noise is input, and the generated data are obtained via adversarial learning between the generator and the discriminator. This entire process is unsupervised, which means that the specific attributes of the generated data cannot be controlled. The key innovation of CGAN lies in the simultaneous introduction of conditional variables into both the generator and the discriminator, optimizing a conditionally constrained loss function. This enables directed control over specific attributes of the generated data, thus producing data that satisfy the desired requirements.

DCGAN builds upon the GAN framework by replacing both the generator and the discriminator with deep convolutional neural networks, which improves the quality of the generated samples and training stability [21,36]; however, it cannot control specific attributes of the generated data. CDCGAN [23,43] is a generative method that merges the strengths of DCGAN and CGAN. Based on DCGAN, it introduces conditional variables to impose generation constraints, thereby enhancing the quality of the generated data.

**Figure 10:**
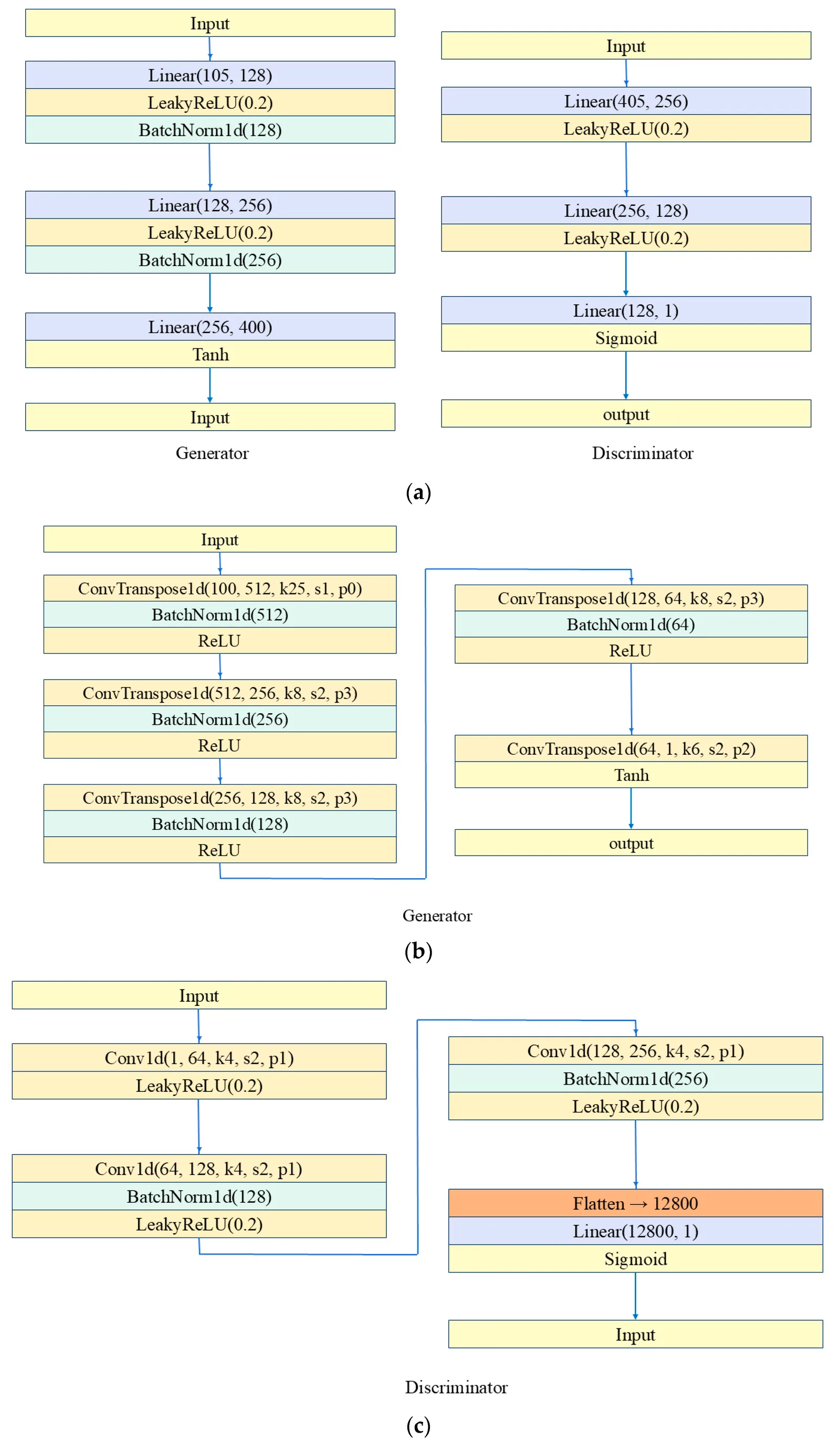

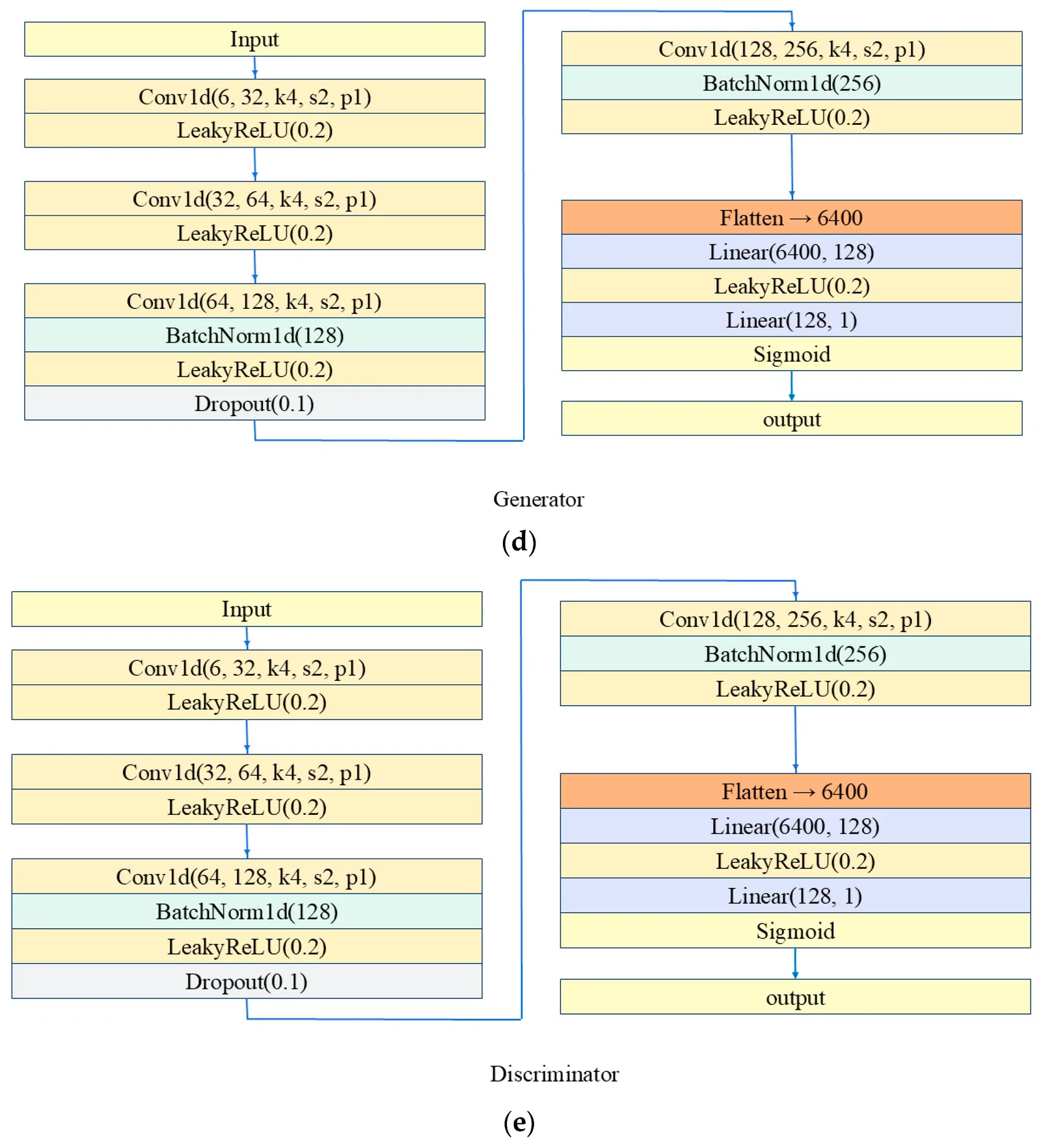
Architectures of the spectral generation models for melon leaves at different disease severity levels for Zhetian 105 and Zhetian 501: (**a**) CGAN; (**b**) generator of DCGAN; (**c**) discriminator of DCGAN; (**d**) generator of CDCGAN; (**e**) discriminator of CDCGAN.

In this study, CGAN, DCGAN, and CDCGAN were employed to generate spectral data of melon leaves at different disease severity levels. Figure 10 illustrates the architectures of the generator and discriminator models for the two varieties. For both melon varieties, the models and parameters used to generate spectra of different disease severity levels were the same. For CGAN, DCGAN, and CDCGAN of the two varieties, the detailed hyperparameters are presented in Table S7. To better generate the spectra, a SAM loss was added in the loss of discriminator, following the research [33]. For each class, the number of generated samples were 100, 200, and 300, respectively, to evaluate the influence of the number of generated samples. The parameter settings of CGAN, DCGAN, and CDCGAN for Zhetian105 and Zhetian 501 are presented in Table S7.

### 4.6. Software, Hardware, and Models

Accuracy, macro F1-Score/precision/recall were used to evaluate the model performance. The LR and SVC models and t-SNE were conducted on Scikit-learn (version 1.7.1), and XGBoost was conducted using XGBoost Python Package (version 3.2.0). All programming was implemented in PyCharm 2023.1 (Python 3.8.19). All deep learning models were conducted on PyTorch 1.12.1. The computer was equipped with an 11th Gen Intel Core i7-11700 CPU and an NVIDIA GeForce RTX 3060 GPU. All models were trained and using the training set, the test set was used to evaluate the model, and the test set was not involved in the model training.

## 5. Conclusions

In this study, hyperspectral imaging was employed to detect the severity of powdery mildew infection on leaves of two melon varieties, Zhetian 105 and Zhetian 501. Classification models using real training samples and real test samples showed the possibility to classify the five disease levels. The spectra generated by CGAN showed noises, and the spectra generated by DCGAN and CDCGAN showed good quality by spectra visual inspection, t-SNE, SAM, WD, and RMSE analysis. The models using generated spectra and the combination of real training samples with generated spectra showed improvements on the prediction of real test samples compared with the corresponding models built using real training samples. Classification models using different numbers of generated spectra showed no specific patterns on the improvement of the prediction of real samples, and the same phenomenon went for the models using the combination of real training samples with different numbers of generated spectra. Overall, different classification models performed differently, and the CNN models and SVC models obtained relatively better performances. Class-wise analysis of real test samples of the SVC models indicated further improvement on data generation of different disease stress levels.

The results in this study illustrated the feasibility of DCGAN and CDCGAN to generate samples of different disease stress levels to improve the prediction performance. Further expansion of the real sample set is needed to generate the representative and diversity samples and more thoroughly evaluate the quality of the generated data. The external validation of the generated data based on unknown real samples should also be further investigated. Future work should also focus on optimizing and improving GAN algorithms to generate data of even higher quality, diversity, and diffusibility.

## Figures and Tables

**Table 1:** The numbers of real samples in the training and test sets of Zhetian 105 and Zhetian 501.

Variety	Dataset	Class				
		0	1	2	3	4
Zhetian 105	Training	26	23	28	19	25
	Test	25	23	28	19	25
Zhetian 501	Training	24	43	17	8	25
	Test	24	43	16	9	25

**Table 2:** Classification results of the models established using real training set samples to predict the real test samples.

Variety	Classifier	Train				Test			
		Accuracy	Precision	F1-Score	Recall	Accuracy	Precision	F1-Score	Recall
Zhetian 105	SVC	79.34%	79.85%	78.94%	79.54%	63.33%	67.13%	64.37%	63.61%
	LR	52.07%	41.27%	44.85%	49.43%	48.33%	37.64%	40.84%	45.77%
	XGBoost	65.29%	65.53%	63.85%	65.01%	54.17%	53.49%	51.79%	54.17%
	CNN	86.78%	86.76%	86.60%	86.51%	64.17%	65.12%	63.94%	64.01%
Zhetian 501	SVC	95.73%	95.70%	95.43%	95.64%	70.09%	55.48%	57.40%	60.19%
	LR	56.41%	26.58%	30.84%	38.73%	56.41%	27.69%	31.36%	38.73%
	XGBoost	73.50%	61.56%	59.57%	60.67%	56.41%	45.21%	46.77%	49.88%
	CNN	89.74%	86.01%	82.84%	82.82%	80.34%	77.11%	73.92%	73.86%

**Table 3:** Classification results of the different classification models using different numbers of generated samples as the training set to predict the real test samples for Zhetian 105.

Method	Number	Classifier	Training				Test			
			Accuracy	Precision	F1-Score	Recall	Accuracy	Precision	F1-Score	Recall
CDCGAN	100	SVC	100.00%	100.00%	100.00%	100.00%	70.83%	74.23%	69.59%	71.89%
		LR	66.80%	66.76%	64.96%	66.80%	57.50%	56.35%	55.25%	58.13%
		XGBoost	96.00%	96.26%	96.00%	96.00%	57.50%	56.87%	56.80%	58.44%
		CNN	98.00%	98.05%	97.99%	98.00%	65.83%	68.53%	65.28%	65.39%
	200	SVC	99.80%	99.80%	99.80%	99.80%	71.67%	76.74%	69.51%	71.36%
		LR	74.80%	74.46%	74.25%	74.80%	60.83%	62.59%	58.41%	60.14%
		XGBoost	97.30%	97.36%	97.31%	97.30%	67.50%	65.88%	65.02%	66.96%
		CNN	99.10%	99.11%	99.10%	99.10%	70.00%	72.27%	67.79%	70.37%
	300	SVC	100.00%	100.00%	100.00%	100.00%	70.00%	70.69%	69.01%	70.16%
		LR	66.13%	67.09%	65.68%	66.13%	61.67%	61.30%	60.38%	61.55%
		XGBoost	97.33%	97.35%	97.34%	97.33%	70.00%	69.62%	68.60%	69.82%
		CNN	99.67%	99.67%	99.67%	99.67%	66.67%	67.04%	66.32%	66.85%
DCGAN	100	SVC	100.00%	100.00%	100.00%	100.00%	70.83%	70.73%	70.00%	70.92%
		LR	63.60%	63.28%	62.52%	63.60%	54.17%	54.83%	52.72%	55.07%
		XGBoost	96.20%	96.31%	96.17%	96.20%	60.00%	59.42%	59.50%	60.21%
		CNN	99.20%	99.23%	99.20%	99.20%	63.33%	64.00%	59.90%	61.16%
	200	SVC	99.80%	99.80%	99.80%	99.80%	70.83%	72.56%	68.40%	68.68%
		LR	64.50%	64.09%	63.51%	64.50%	55.83%	56.85%	55.18%	56.34%
		XGBoost	97.60%	97.65%	97.58%	97.60%	62.50%	63.17%	62.38%	62.51%
		CNN	100.00%	100.00%	100.00%	100.00%	70.00%	69.52%	63.28%	66.47%
	300	SVC	99.87%	99.87%	99.87%	99.87%	70.00%	72.44%	67.49%	67.88%
		LR	64.80%	64.15%	63.81%	64.80%	58.33%	59.85%	58.10%	58.49%
		XGBoost	98.73%	98.74%	98.73%	98.73%	68.33%	67.38%	67.30%	68.12%
		CNN	100.00%	100.00%	100.00%	100.00%	66.67%	67.85%	62.62%	63.85%

**Table 4:** Classification results of the different classification models using real training samples with different numbers of generated samples as training set to predict the real test set samples for Zhetian 105.

Method	Number	Classifier	Training				Test			
			Accuracy	Precision	F1-Score	Recall	Accuracy	Precision	F1-Score	Recall
CDCGAN	100	SVC	98.23%	98.22%	98.23%	98.25%	70.83%	72.92%	69.97%	71.11%
		LR	66.34%	66.62%	65.41%	66.56%	56.67%	55.54%	54.83%	57.18%
		XGBoost	95.01%	95.08%	95.01%	95.02%	62.50%	63.19%	62.40%	63.12%
		CNN	97.10%	97.09%	97.09%	97.13%	68.33%	71.09%	66.96%	68.13%
	200	SVC	99.11%	99.11%	99.11%	99.11%	75.00%	76.56%	74.22%	74.84%
		LR	71.28%	70.73%	70.71%	71.27%	63.33%	64.18%	61.48%	62.60%
		XGBoost	96.70%	96.79%	96.72%	96.70%	72.50%	72.30%	71.19%	71.87%
		CNN	98.39%	98.41%	98.40%	98.40%	68.33%	71.08%	68.16%	68.70%
	300	SVC	99.14%	99.13%	99.14%	99.14%	70.00%	71.89%	69.67%	69.88%
		LR	65.70%	66.39%	65.34%	65.78%	61.67%	61.30%	60.38%	61.55%
		XGBoost	96.85%	96.86%	96.85%	96.86%	70.00%	69.44%	68.99%	69.98%
		CNN	98.77%	98.78%	98.76%	98.76%	70.83%	71.29%	69.49%	69.58%
DCGAN	100	SVC	98.55%	98.54%	98.55%	98.55%	71.67%	72.03%	71.26%	71.63%
		LR	60.87%	60.97%	60.64%	61.05%	56.67%	57.34%	56.23%	56.90%
		XGBoost	96.30%	96.31%	96.29%	96.30%	62.50%	62.78%	62.27%	63.00%
		CNN	97.26%	97.32%	97.28%	97.26%	67.50%	67.14%	66.40%	66.90%
	200	SVC	98.93%	98.94%	98.93%	98.92%	69.17%	70.51%	67.86%	67.66%
		LR	63.78%	63.39%	63.07%	63.92%	56.67%	57.34%	56.23%	56.90%
		XGBoost	97.15%	97.13%	97.13%	97.16%	63.33%	62.92%	62.83%	63.40%
		CNN	98.48%	98.50%	98.49%	98.50%	69.17%	70.69%	69.18%	69.67%
	300	SVC	99.26%	99.27%	99.26%	99.26%	69.17%	69.54%	67.64%	67.66%
		LR	64.22%	63.66%	63.44%	64.31%	58.33%	59.85%	58.10%	58.49%
		XGBoost	98.33%	98.33%	98.33%	98.34%	68.33%	67.36%	67.12%	68.21%
		CNN	99.63%	99.63%	99.63%	99.63%	67.50%	68.60%	67.01%	67.48%

**Table 5:** Classification results of the different classification models using different numbers of generated samples as the training set to predict the real test set samples for Zhetian 501.

Method	Number	Classifier	Training				Test			
			Accuracy	Precision	F1-Score	Recall	Accuracy	Precision	F1-Score	Recall
CDCGAN	100	SVC	100.00%	100.00%	100.00%	100.00%	76.07%	73.41%	73.40%	77.90%
		LR	60.00%	62.26%	57.84%	60.00%	65.81%	68.43%	62.94%	68.35%
		XGBoost	98.40%	98.40%	98.40%	98.40%	65.81%	58.29%	57.93%	60.08%
		CNN	100.00%	100.00%	100.00%	100.00%	74.36%	63.83%	64.05%	64.79%
	200	SVC	100.00%	100.00%	100.00%	100.00%	77.78%	76.69%	76.60%	77.02%
		LR	70.80%	74.09%	69.60%	70.80%	75.21%	77.56%	71.35%	72.27%
		XGBoost	99.30%	99.31%	99.30%	99.30%	65.81%	56.84%	56.65%	58.35%
		CNN	100.00%	100.00%	100.00%	100.00%	81.20%	77.33%	77.31%	79.01%
	300	SVC	100.00%	100.00%	100.00%	100.00%	78.63%	74.84%	76.09%	78.66%
		LR	77.07%	79.43%	76.53%	77.07%	66.67%	58.15%	55.77%	59.02%
		XGBoost	99.60%	99.60%	99.60%	99.60%	64.10%	55.04%	55.63%	56.69%
		CNN	99.93%	99.93%	99.93%	99.93%	70.09%	69.71%	66.20%	67.03%
DCGAN	100	SVC	98.00%	98.03%	98.00%	98.00%	82.05%	75.55%	76.16%	77.83%
		LR	56.00%	56.61%	52.40%	56.00%	65.81%	68.90%	63.55%	66.78%
		XGBoost	98.20%	98.20%	98.19%	98.20%	70.09%	63.39%	63.86%	65.21%
		CNN	98.60%	98.69%	98.60%	98.60%	81.20%	76.69%	74.93%	77.18%
	200	SVC	99.90%	99.90%	99.90%	99.90%	79.49%	72.62%	72.79%	74.34%
		LR	60.10%	60.66%	57.89%	60.10%	70.09%	69.25%	68.27%	70.53%
		XGBoost	98.60%	98.62%	98.60%	98.60%	67.52%	60.78%	61.14%	61.87%
		CNN	99.80%	99.80%	99.80%	99.80%	81.20%	73.38%	70.71%	70.89%
	300	SVC	99.87%	99.87%	99.87%	99.87%	77.78%	70.78%	70.85%	72.81%
		LR	61.13%	61.12%	59.00%	61.13%	71.79%	70.57%	71.27%	74.97%
		XGBoost	98.93%	98.95%	98.93%	98.93%	68.38%	61.81%	61.45%	61.92%
		CNN	98.13%	98.28%	98.14%	98.13%	81.20%	76.89%	70.42%	75.01%

**Table 6:** Classification results of the different classification models using real training samples with different numbers of generated samples as the training set to predict the real test set samples for Zhetian 501.

Method	Number	Classifier	Training				Test			
			Accuracy	Precision	F1-Score	Recall	Accuracy	Precision	F1-Score	Recall
CDCGAN	100	SVC	97.73%	97.70%	97.74%	97.82%	76.07%	66.29%	66.73%	68.14%
		LR	52.35%	52.12%	44.31%	50.71%	68.38%	68.22%	57.56%	59.17%
		XGBoost	97.24%	97.21%	97.26%	97.33%	69.23%	60.97%	61.33%	62.35%
		CNN	98.70%	98.76%	98.75%	98.74%	83.76%	78.65%	79.18%	80.78%
	200	SVC	98.57%	98.58%	98.58%	98.60%	83.76%	78.62%	78.41%	79.02%
		LR	67.23%	72.79%	64.83%	66.67%	75.21%	70.78%	66.41%	68.16%
		XGBoost	98.30%	98.30%	98.31%	98.34%	68.38%	60.89%	61.23%	63.07%
		CNN	99.46%	99.45%	99.47%	99.49%	85.47%	83.21%	83.05%	83.85%
	300	SVC	98.95%	98.94%	98.95%	98.97%	83.76%	79.79%	81.18%	83.84%
		LR	75.14%	78.02%	74.43%	74.86%	66.67%	56.19%	53.91%	57.27%
		XGBoost	98.64%	98.64%	98.64%	98.64%	60.68%	49.89%	50.63%	51.92%
		CNN	99.13%	99.13%	99.12%	99.12%	81.20%	75.54%	74.95%	75.21%
DCGAN	100	SVC	96.11%	96.03%	96.10%	96.28%	73.50%	68.41%	69.41%	71.00%
		LR	53.16%	54.79%	45.41%	51.50%	68.38%	67.86%	57.72%	58.80%
		XGBoost	97.89%	97.88%	97.95%	98.04%	69.23%	62.86%	63.26%	64.74%
		CNN	97.57%	97.49%	97.56%	97.67%	82.91%	78.90%	79.31%	80.36%
	200	SVC	99.02%	99.01%	99.00%	99.00%	76.07%	69.18%	69.48%	70.62%
		LR	58.28%	59.63%	54.70%	57.65%	71.79%	68.76%	68.16%	68.74%
		XGBoost	98.75%	98.74%	98.76%	98.80%	66.67%	60.13%	60.53%	61.41%
		CNN	97.58%	97.71%	97.51%	97.46%	82.05%	75.35%	75.48%	76.12%
	300	SVC	99.38%	99.38%	99.38%	99.38%	80.34%	73.71%	74.07%	75.04%
		LR	61.29%	61.52%	58.66%	60.95%	73.50%	71.25%	71.82%	73.18%
		XGBoost	98.83%	98.83%	98.84%	98.87%	70.94%	64.30%	63.52%	64.02%
		CNN	99.57%	99.56%	99.57%	99.59%	83.76%	77.72%	78.10%	79.39%

**Table 7:** Sample statistics of melon leaves of varieties Zhetian 105 and Zhetian 501 under different disease severity levels.

Disease Severity	Zhetian 105	Zhetian 501
Healthy CK	51	48
Level 1	46	86
Level 2	56	33
Level 3	38	17
Level 4	50	50

## Data Availability

The original contributions presented in this study are included in the article. Further inquiries can be directed to the corresponding author.

## Supplementary Materials

The following supporting information can be downloaded at: https://www.mdpi.com/article/10.3390/plants15172639/s1, Table S1: Average values of spectral map angle (SAM), Wasserstein distance (WD), and root mean square error (RMSE) of the generated sample with real training samples for Zhetian 105. Table S2: Average values of spectral map angle (SAM), Wasserstein distance (WD), and root mean square error (RMSE) of the generated sample with real training samples for Zhetian 501; Table S3: Class-wise prediction results of the real test samples by all SVC models of Zhetian 105; Table S4: Class-wise prediction results of the real test samples by all SVC models of Zhetian 501; Table S5: Criteria for grading the severity of powdery mildew on melon leaves; Table S6: The hyperparameters determined by three-fold cross-validation of CNN models; Table S7: Parameter settings of CGAN, DCGAN, and CDCGAN for Zhetian105 and Zhetian 501; Figure S1: Comparison of representative leaves of Zhetian 105 melon cultivar at different disease severity levels: (a) healthy CK; (b) disease severity level 1; (c) disease severity level 2; (d) disease severity level 3; (e) disease severity level 4; Figure S2: Comparison of representative leaves of Zhetian 501 melon cultivar at different disease severity levels: (a) healthy CK; (b) disease severity level 1; (c) disease severity level 2; (d) disease severity level 3; (e) disease severity level 4.
