# Supplementary material for: Application of Hyperspectral Imaging and Generative Adversarial Network for Powdery Mildew Severity Detection on Melon Leaves
Source: Plants (Basel). 2026 Aug 28;15(17):2639. doi: 10.3390/plants15172639 (PMC13567362; doi:10.3390/plants15172639)
Supplement: Supplementary file 1 [file plants-15-02639-s001.zip › plants-4378258-supplementary.pdf]

Supplementary Materials

Table S1 Average values of Spectral map angle (SAM), Wasserstein distance (WD) and Root mean square error (RMSE) of the generated sample with real training samples for Zhetian 105

Model	Generated number	Class	SAM (rad)	WD	RMSE
CDCGAN	100	0	0.01082855	0.00898619	0.01106919
CDCGAN	100	1	0.00802166	0.00383990	0.00254224
CDCGAN	100	2	0.01131571	0.00681570	0.00366704
CDCGAN	100	3	0.01060583	0.00738427	0.00768672
CDCGAN	100	4	0.00923068	0.00432432	0.00277376
CDCGAN	200	0	0.01150849	0.00523453	0.00553438
CDCGAN	200	1	0.00992327	0.01341525	0.01770493
CDCGAN	200	2	0.01124962	0.00402699	0.00229495
CDCGAN	200	3	0.00985581	0.00695721	0.00103807
CDCGAN	200	4	0.00772971	0.00533543	0.00314938
CDCGAN	300	0	0.01111906	0.00439096	0.00280026
CDCGAN	300	1	0.00860101	0.00560672	0.00638326
CDCGAN	300	2	0.01105653	0.00510690	0.00477062
CDCGAN	300	3	0.00980913	0.00680869	0.00233628
CDCGAN	300	4	0.00789900	0.00595007	0.00564909
DCGAN	100	0	0.01112143	0.00488254	0.00062922
DCGAN	100	1	0.01028995	0.00321321	0.00209470
DCGAN	100	2	0.01332789	0.00426504	0.00151426
DCGAN	100	3	0.01485011	0.00957281	0.00966402
DCGAN	100	4	0.01027889	0.00421483	0.00184910
DCGAN	200	0	0.01156786	0.00503890	0.00089200
DCGAN	200	1	0.00937568	0.00318663	0.00161937
DCGAN	200	2	0.01430270	0.00424299	0.00091149
DCGAN	200	3	0.01419892	0.00820734	0.00858759
DCGAN	200	4	0.01075138	0.00413383	0.00202402
DCGAN	300	0	0.01192546	0.00501040	0.00075073
DCGAN	300	1	0.00954378	0.00298375	0.00151991
DCGAN	300	2	0.01389571	0.00385178	0.00107538
DCGAN	300	3	0.01435217	0.00739416	0.00743927
DCGAN	300	4	0.01051237	0.00406327	0.00216965

Table S2 Average values of Spectral map angle (SAM), Wasserstein distance (WD) and Root mean square error (RMSE) of the generated sample with real training samples for Zhetian 501

Model	Generated number	Class	SAM(rad)	WD	RMSE
CDCGAN	100	0	0.00814976	0.00725273	0.00839316
CDCGAN	100	1	0.01044445	0.00880595	0.00695499

Model	Generated number	Class	SAM(rad)	WD	RMSE
CDCGAN	100	2	0.01041885	0.00771037	0.00883273
CDCGAN	100	3	0.01385518	0.00947702	0.00871697
CDCGAN	100	4	0.01069291	0.00906409	0.00821463
CDCGAN	200	0	0.00829648	0.00553681	0.00350311
CDCGAN	200	1	0.01152559	0.00701484	0.00229344
CDCGAN	200	2	0.00998383	0.00976852	0.00976819
CDCGAN	200	3	0.01522750	0.00575440	0.00292961
CDCGAN	200	4	0.01274389	0.00522132	0.00362971
CDCGAN	300	0	0.00767462	0.00601750	0.00644686
CDCGAN	300	1	0.01246799	0.04243306	0.04899696
CDCGAN	300	2	0.00906912	0.00711771	0.00684207
CDCGAN	300	3	0.01664203	0.00956550	0.00737268
CDCGAN	300	4	0.01441576	0.00857051	0.00679292
DCGAN	100	0	0.00785417	0.00217410	0.00046318
DCGAN	100	1	0.01492688	0.00791771	0.00462974
DCGAN	100	2	0.02380994	0.00270142	0.00150180
DCGAN	100	3	0.02492822	0.00404551	0.00141438
DCGAN	100	4	0.01692859	0.00478216	0.00392800
DCGAN	200	0	0.00771762	0.00237341	0.00128973
DCGAN	200	1	0.01463997	0.00727404	0.00391912
DCGAN	200	2	0.02375056	0.00251848	0.00121470
DCGAN	200	3	0.02401304	0.00298127	0.00106350
DCGAN	200	4	0.01615581	0.00479249	0.00262290
DCGAN	300	0	0.00779895	0.00296161	0.00161926
DCGAN	300	1	0.01466497	0.00586711	0.00221428
DCGAN	300	2	0.02340684	0.00268527	0.00115842
DCGAN	300	3	0.02327528	0.00308051	0.00122043
DCGAN	300	4	0.01649336	0.00528833	0.00343605

Table S3 Class-wise prediction results of the real test samples by all SVC models of Zhetian 105

Training sample	generative model	Number	Category	Real test samples results			
				Accuracy	Precision	Recall	F1-score
Real training samples	--	--	0	68.00%	89.47%	68.00%	77.27%
			1	60.87%	50.00%	60.87%	54.90%
			2	50.00%	50.00%	50.00%	50.00%
			3	63.16%	46.15%	63.16%	53.33%
			4	76.00%	100.00%	76.00%	86.36%
Generated	CDCGAN	100	0	96.00%	77.42%	96.00%	85.71%

training samples			1	39.13%	81.82%	39.13%	52.94%
			2	53.57%	71.43%	53.57%	61.22%
			3	94.74%	50.00%	94.74%	65.45%
			4	76.00%	90.48%	76.00%	82.61%
			0	96.00%	72.73%	96.00%	82.76%
			1	30.43%	100.00%	30.43%	46.67%
		200	2	71.43%	64.52%	71.43%	67.80%
			3	78.95%	55.56%	78.95%	65.22%
			4	80.00%	90.91%	80.00%	85.11%
			0	92.00%	88.46%	92.00%	90.20%
			1	39.13%	69.23%	39.13%	50.00%
		300	2	60.71%	60.71%	60.71%	60.71%
			3	78.95%	51.72%	78.95%	62.50%
			4	80.00%	83.33%	80.00%	81.63%
			0	96.00%	85.71%	96.00%	90.57%
			1	52.17%	75.00%	52.17%	61.54%
		100	2	50.00%	58.33%	50.00%	53.85%
			3	68.42%	50.00%	68.42%	57.78%
			4	88.00%	84.62%	88.00%	86.27%
			0	96.00%	88.89%	96.00%	92.31%
			1	39.13%	81.82%	39.13%	52.94%
	DCGAN	200	2	82.14%	54.76%	82.14%	65.71%
			3	42.11%	53.33%	42.11%	47.06%
			4	84.00%	84.00%	84.00%	84.00%
			0	96.00%	82.76%	96.00%	88.89%
			1	39.13%	90.00%	39.13%	54.55%
		300	2	82.14%	56.10%	82.14%	66.67%
			3	42.11%	50.00%	42.11%	45.71%
			4	80.00%	83.33%	80.00%	81.63%
			0	96.00%	82.76%	96.00%	88.89%
			1	43.48%	83.33%	43.48%	57.14%
		100	2	57.14%	61.54%	57.14%	59.26%
			3	78.95%	50.00%	78.95%	61.22%
			4	80.00%	86.96%	80.00%	83.33%
			0	96.00%	82.76%	96.00%	88.89%
Real and			1	47.83%	84.62%	47.83%	61.11%
generated	CDCGAN	200	2	71.43%	64.52%	71.43%	67.80%
samples			3	78.95%	60.00%	78.95%	68.18%
			4	80.00%	90.91%	80.00%	85.11%
			0	96.00%	92.31%	96.00%	94.12%
			1	47.83%	78.57%	47.83%	59.46%
		300	2	57.14%	55.17%	57.14%	56.14%
			3	68.42%	46.43%	68.42%	55.32%
			4	80.00%	86.96%	80.00%	83.33%

			0	96.00%	96.00%	96.00%	96.00%
			1	52.17%	70.59%	52.17%	60.00%
		100	2	53.57%	55.56%	53.57%	54.55%
			3	68.42%	50.00%	68.42%	57.78%
			4	88.00%	88.00%	88.00%	88.00%
			0	96.00%	92.31%	96.00%	94.12%
			1	43.48%	76.92%	43.48%	55.56%
	DCGAN	200	2	71.43%	52.63%	71.43%	60.61%
			3	47.37%	47.37%	47.37%	47.37%
			4	80.00%	83.33%	80.00%	81.63%
			0	96.00%	88.89%	96.00%	92.31%
			1	43.48%	71.43%	43.48%	54.05%
		300	2	71.43%	54.05%	71.43%	61.54%
			3	47.37%	50.00%	47.37%	48.65%
			4	80.00%	83.33%	80.00%	81.63%

Table S4 Class-wise prediction results of the real test samples by all SVC models of Zhetian 501

Training sample	generative model	Number	Category	Real test samples results			
				Accuracy	Precision	Recall	F1-score
			0	87.50%	72.41%	87.50%	79.25%
			1	67.44%	80.56%	67.44%	73.42%
Real training samples	--	--	2	50.00%	44.44%	50.00%	47.06%
			3	0.00%	0.00%	0.00%	0.00%
			4	96.00%	80.00%	96.00%	87.27%
			0	100.00%	68.57%	100.00%	81.36%
			1	55.81%	100.00%	55.81%	71.64%
		100	2	75.00%	60.00%	75.00%	66.67%
			3	66.67%	50.00%	66.67%	57.14%
			4	92.00%	88.46%	92.00%	90.20%
			0	83.33%	66.67%	83.33%	74.07%
			1	72.09%	79.49%	72.09%	75.61%
Generated training samples	CDCGAN	200	2	75.00%	75.00%	75.00%	75.00%
			3	66.67%	66.67%	66.67%	66.67%
			4	88.00%	95.65%	88.00%	91.67%
			0	95.83%	76.67%	95.83%	85.19%
			1	67.44%	87.88%	67.44%	76.32%
		300	2	56.25%	50.00%	56.25%	52.94%
			3	77.78%	63.64%	77.78%	70.00%
			4	96.00%	96.00%	96.00%	96.00%
	DCGAN	100	0	100.00%	80.00%	100.00%	88.89%
			1	79.07%	97.14%	79.07%	87.18%

			2	62.50%	66.67%	62.50%	64.52%
			3	55.56%	45.45%	55.56%	50.00%
			4	92.00%	88.46%	92.00%	90.20%
			0	100.00%	75.00%	100.00%	85.71%
			1	76.74%	97.06%	76.74%	85.71%
		200	2	62.50%	66.67%	62.50%	64.52%
			3	44.44%	36.36%	44.44%	40.00%
			4	88.00%	88.00%	88.00%	88.00%
			0	100.00%	77.42%	100.00%	87.27%
			1	76.74%	91.67%	76.74%	83.54%
		300	2	43.75%	58.33%	43.75%	50.00%
			3	55.56%	38.46%	55.56%	45.45%
			4	88.00%	88.00%	88.00%	88.00%
			0	95.83%	85.19%	95.83%	90.20%
			1	74.42%	94.12%	74.42%	83.12%
		100	2	56.25%	45.00%	56.25%	50.00%
			3	22.22%	25.00%	22.22%	23.53%
			4	92.00%	82.14%	92.00%	86.79%
			0	100.00%	82.76%	100.00%	90.57%
			1	81.40%	92.11%	81.40%	86.42%
	CDCGAN	200	2	81.25%	76.47%	81.25%	78.79%
			3	44.44%	57.14%	44.44%	50.00%
			4	88.00%	84.62%	88.00%	86.27%
			0	100.00%	82.76%	100.00%	90.57%
			1	74.42%	91.43%	74.42%	82.05%
		300	2	75.00%	70.59%	75.00%	72.73%
			3	77.78%	58.33%	77.78%	66.67%
Real and generated samples			4	92.00%	95.83%	92.00%	93.88%
			0	79.17%	73.08%	79.17%	76.00%
			1	69.77%	83.33%	69.77%	75.95%
		100	2	62.50%	55.56%	62.50%	58.82%
			3	55.56%	45.45%	55.56%	50.00%
			4	88.00%	84.62%	88.00%	86.27%
			0	91.67%	75.86%	91.67%	83.02%
			1	76.74%	89.19%	76.74%	82.50%
	DCGAN	200	2	56.25%	60.00%	56.25%	58.06%
			3	44.44%	33.33%	44.44%	38.10%
			4	84.00%	87.50%	84.00%	85.71%
			0	91.67%	84.62%	91.67%	88.00%
			1	83.72%	90.00%	83.72%	86.75%
		300	2	56.25%	64.29%	56.25%	60.00%
			3	55.56%	41.67%	55.56%	47.62%
			4	88.00%	88.00%	88.00%	88.00%

Table S5 Criteria for grading the severity of powdery mildew on melon leaves

Disease Severity Level	Symptom Description
0	No symptoms
1	White spots appear on the upper leaf surface, sometimes with white mildew. The spots are scattered and not connected to each other. The total area of lesions accounts for less than 5% of the leaf area.
2	White spots appear on the upper leaf surface, sometimes with white mildew. The spots are scattered over the entire leaf surface and are not connected to each other. The total area of lesions accounts for 5%-25% of the leaf area.
3	A large number of white spots or patches with mildew appear on the upper leaf surface. Some lesions coalesce to form patches. The total area of lesions accounts for 25%-50% of the leaf area.
4	The upper leaf surface is densely covered with white spots or patches, which bear thick mildew and coalesce into large patches. The total area of lesions accounts for more than 50% of the leaf area. Some lesions turn yellowish-brown or even become necrotic.

Table S6 The hyperparameters determined by three-fold cross-validation of CNN models

**Number**	**Methods**	**Variety (Zhetian)**	**Training set**	**Generated number**	**Learning rate**	**BATCH_SIZE**
1	CDCGAN	105	Real training samples	—	0.001	32
2	CDCGAN	105	Generated samples	100	0.001	8
3	CDCGAN	105	Generated samples	200	0.005	8
4	CDCGAN	105	Generated samples	300	0.001	64
5	CDCGAN	105	Real training samples + generated samples	100	0.001	16
6	CDCGAN	105	Real training samples + generated samples	200	0.001	64
7	CDCGAN	105	Real training samples + generated samples	300	0.001	8
8	CDCGAN	501	Real training samples	—	0.005	8
9	CDCGAN	501	Generated samples	100	0.001	4
10	CDCGAN	501	Generated samples	200	0.001	8
11	CDCGAN	501	Generated samples	300	0.001	4
12	CDCGAN	501	Real training samples + generated samples	100	0.001	16
13	CDCGAN	501	Real training samples + generated samples	200	0.001	32
14	CDCGAN	501	Real training samples + generated samples	300	0.001	8
15	DCGAN	105	Generated samples	100	0.001	8
16	DCGAN	105	Generated samples	200	0.001	32
17	DCGAN	105	Generated samples	300	0.001	16

18	DCGAN	105	Real training samples + generated samples	100	0.001	8
19	DCGAN	105	Real training samples + generated samples	200	0.001	64
20	DCGAN	105	Real training samples + generated samples	300	0.001	64
21	DCGAN	501	Generated samples	100	0.001	32
22	DCGAN	501	Generated samples	200	0.001	32
23	DCGAN	501	Generated samples	300	0.001	64
24	DCGAN	501	Real training samples + generated samples	100	0.001	32
25	DCGAN	501	Real training samples + generated samples	200	0.001	4
26	DCGAN	501	Real training samples + generated samples	300	0.001	64

Table S7 Parameter settings of CGAN, DCGAN and CDCGAN for Zhetian105 and Zhetian 501

**Parameters**	**CDCGAN**	**DCGAN**	**CGAN**
Nosie dimension	100	100	100
BATCH_SIZE	32	16	16
Epochs	5001	8001	5001
Learning rate of Generartor	0.0004	0.0002	0.0004
Learning rate of Discriminator	0.0002	0.0002	0.0001
(Adam, betas)	(0.5, 0.999)	(0.5, 0.999)	(0.5, 0.999)
Adversarial Loss	BCELoss + Sigmoid	BCELoss + Sigmoid	BCELoss + Sigmoid
Generator Loss	BCE_adv + SAM	BCE_adv + SAM	BCE_adv + SAM

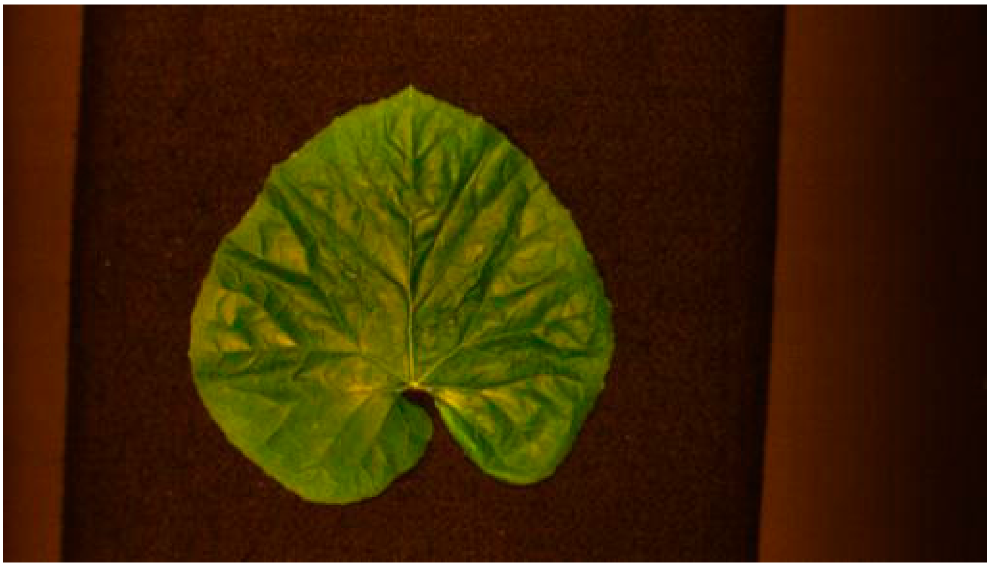

(a)

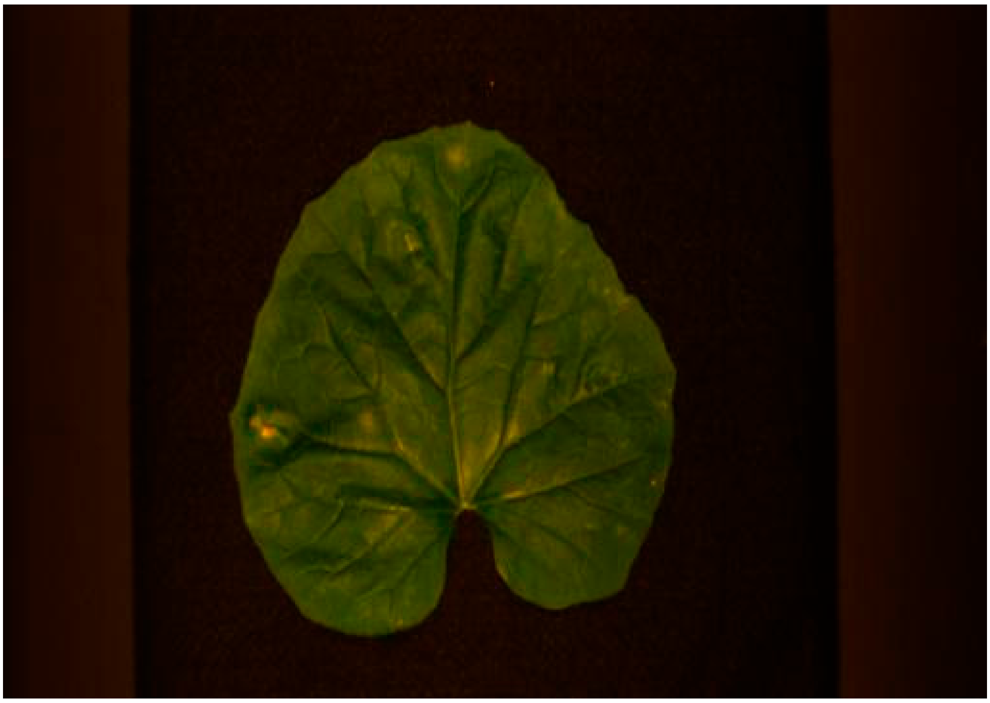

(b)

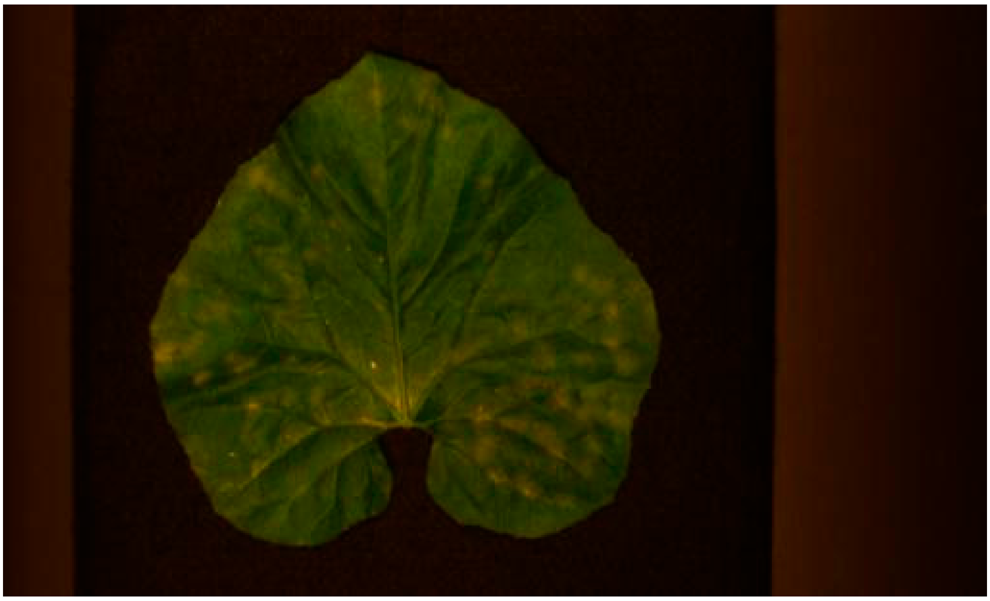

(c)

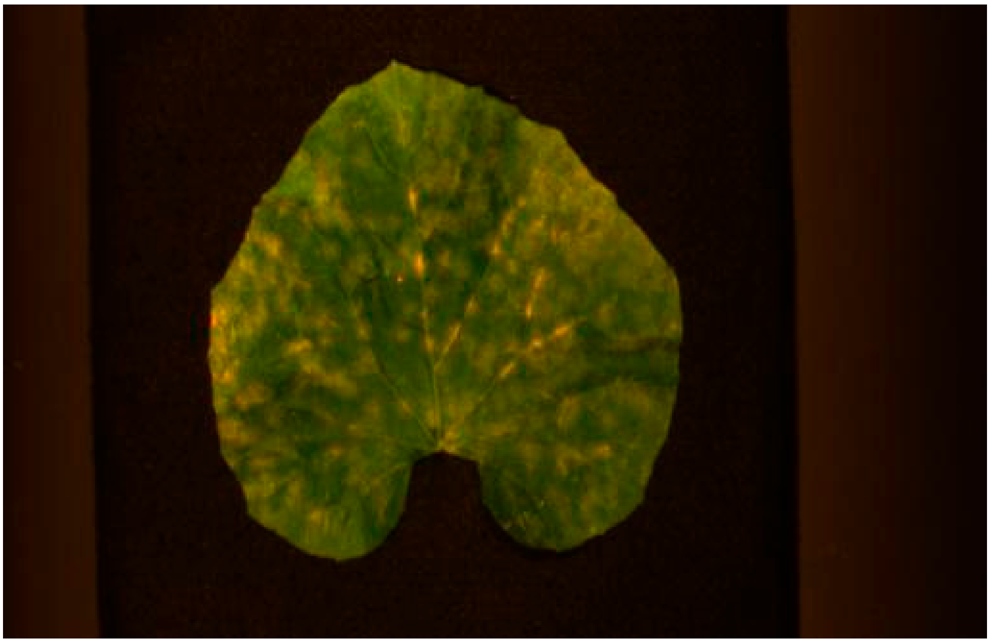

(d)

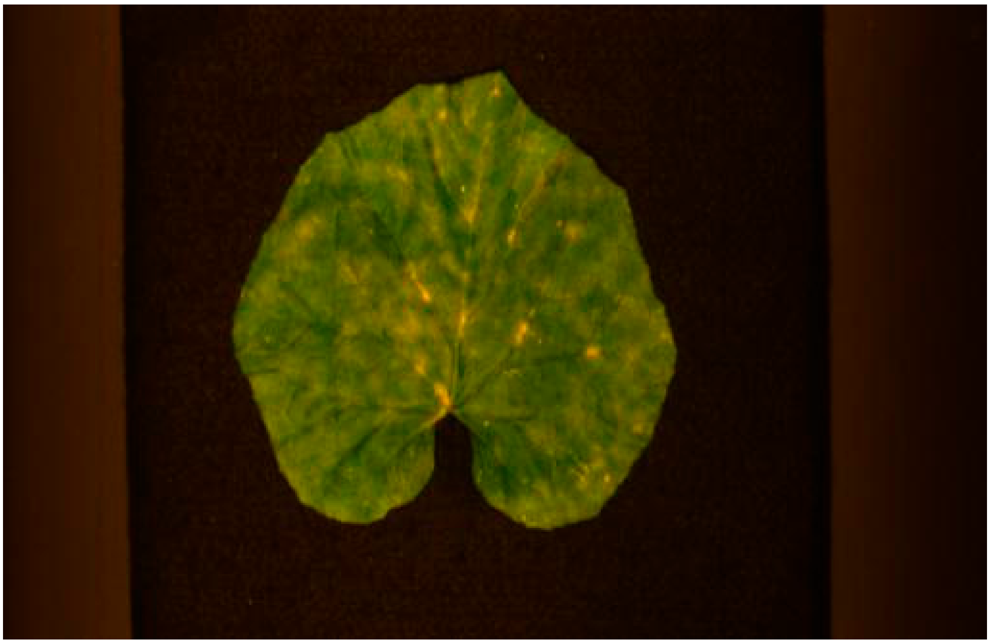

(e)

Figure S1 Comparison of representative leaves of Zhetian 105 melon cultivar at different disease severity levels. (a):Healthy CK; (b): Disease severity Level 1; (c): Disease severity Level 2; (d): Disease severity Level 3;(e): Disease severity Level 4.

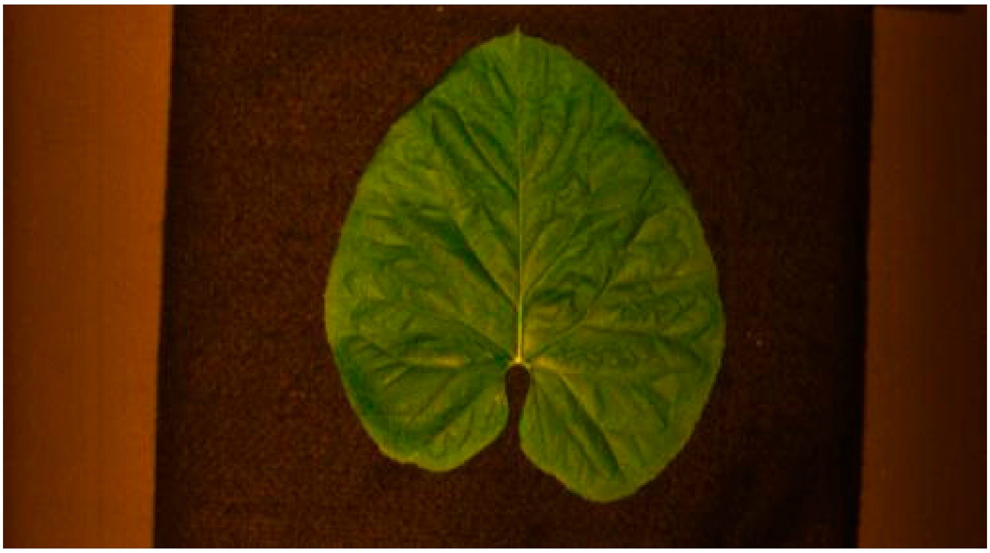

(a)

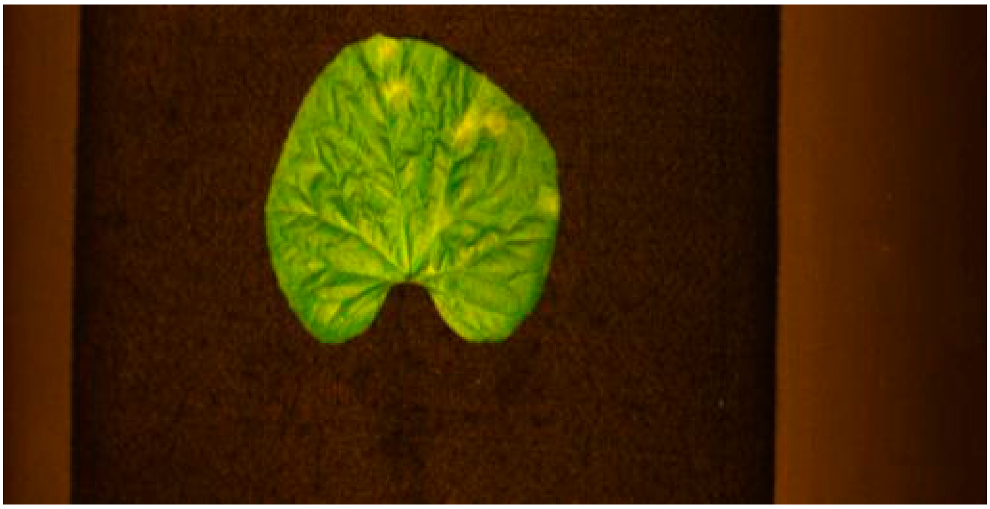
(b)
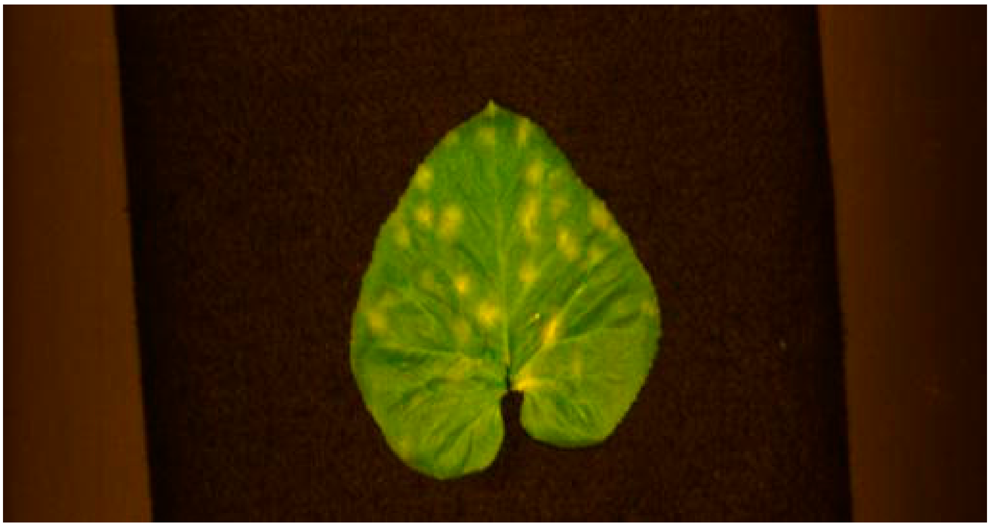
(c)
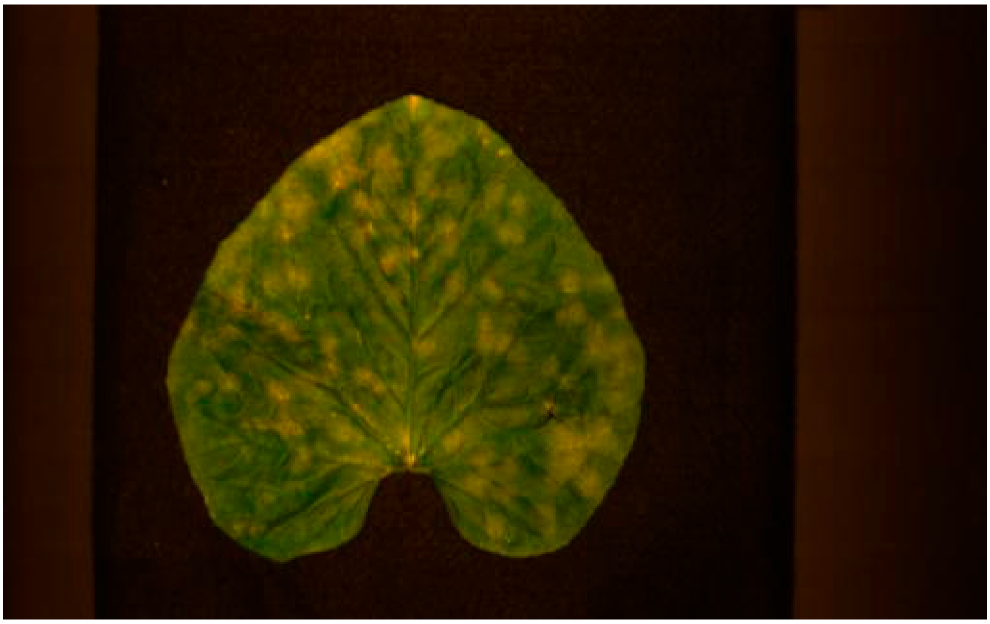
(d)

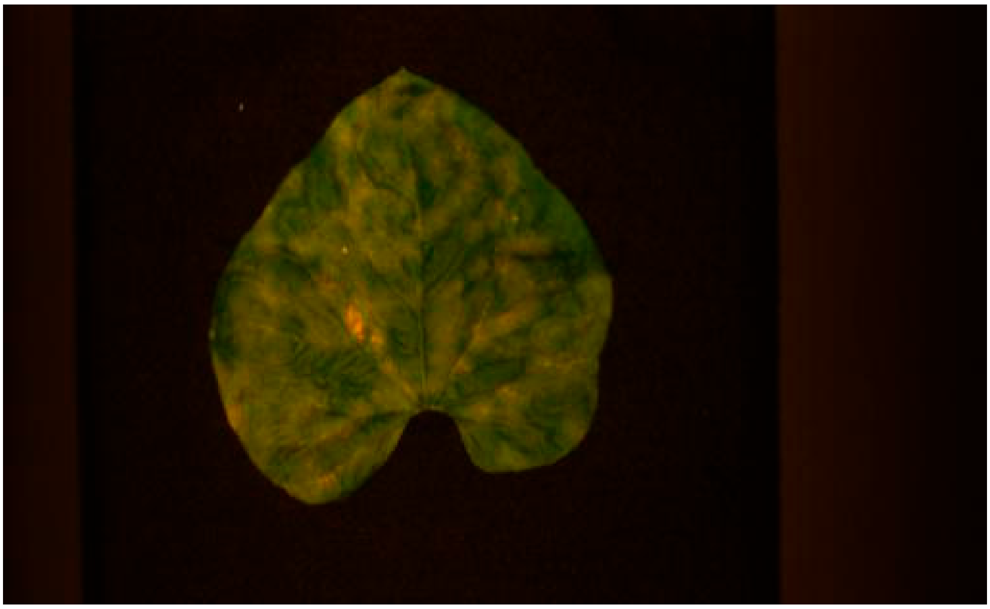

(e)

Figure S2 Comparison of representative leaves of Zhetian 501 melon cultivar at different disease severity levels. (a):Healthy CK; (b): Disease severity Level 1; (c): Disease severity Level 2; (d): Disease severity Level 3;(e): Disease severity Level 4.
